# Pharmacology and macrophage modulation of HPGDS inhibitor PK007 demonstrate reduced disease severity in DMD-affected muscles of the *mdx* mouse model

**DOI:** 10.1186/s13395-025-00379-1

**Published:** 2025-04-24

**Authors:** Sai Yarlagadda, Chynna-Loren Sheremeta, Sang Won Cheung, Alison Cuffe, Miranda D. Grounds, Mark L. Smythe, Peter G. Noakes

**Affiliations:** 1 School of Biomedical Sciences, Faculty of Medicine, the University of Queensland, Brisbane, QLD 4072 Australia; 2 Institute for Molecular Bioscience, the University of Queensland, Brisbane, QLD 4072 Australia; 3 School of Human Biology, the University of Western Australia, Perth, WA 6009 Australia

**Keywords:** HPGDS, PGD2, DMD, Myonecrosis, Macrophage, Muscle strength, Phenomaster, Cytokines, Pro-inflammatory, TNF-α, IL-1β and iNOS

## Abstract

**Background:**

Duchenne Muscular Dystrophy (DMD) is an X-linked disease characterised by chronic inflammation, progressive muscle damage, and muscle loss. Typically, initial symptoms affect lower limb muscles, including the *gastrocnemius* (GA), *tibialis anterior* (TA), and *extensor digitorum longus* (EDL). During the acute phase of DMD, particularly in boys aged 2–8 years, muscle damage resulting in necrosis (myonecrosis) involves a complex immune-inflammatory response. Prostaglandin D2 (PGD2) is recognised for enhancing pro-inflammatory chemokine and interleukin signalling and recruiting infiltrating immune cells such as pro-inflammatory macrophages, exacerbating myonecrosis.

**Methods:**

To reduce levels of PGD2, a novel hematopoietic prostaglandin D2 synthase (HPGDS) inhibitor, PK007, was characterised (i) for potency and pharmacokinetic profiles and then tested in the *mdx* mouse model of DMD during the acute early onset of disease progression. Juvenile *mdx and wild type (WT) C57Bl/10Scsn* mice were orally treated with PK007 and control vehicle solution for 10 days, from postnatal day 18 to 28. This builds upon a previous study with PK007 with (ii) additional analyses of disease progression assessed for muscle grip strength, metabolic and locomotor activity, myonecrosis in a wide range of muscles (3 from hindlimb, diaphragm, heart, and tongue), macrophage infiltration and pro-inflammatory cytokines (TNF-α, IL-1β and iNOS).

**Results:**

PK007 exhibited high potency (17.23 ± 12 nM), a long half-life (3.0 ± 0.3 h), and good oral bioavailability (81%). Treatment with PK007 decreased serum PGD2 levels (33.36%) in *mdx* mice compared to control (vehicle-treated) *mdx* mice. In *mdx* mice (compared with controls), PK007 enhanced grip strength (69.05% increase) and improved locomotor activity (69.05% increase). Histological analysis revealed a significant reduction in the total myonecrotic area in PK007-treated GA (49.75%), TA (73.87%), EDL (60.31%), diaphragm (48.02%), and tongue (37.93%) muscles of *mdx* mice (compared with controls). Additionally, PK007 decreased macrophage cell area by 55.56% in GA and 47.83% in EDL muscles. Further expression of pro-inflammatory cytokines and enzymes such as TNF-α, IL-1β and iNOS were significantly reduced in PK007 treated mice. These results demonstrate that PK007 significantly reduces the inflammatory response, protects muscles from necrosis and increases strength in juvenile *mdx* mice.

**Conclusion:**

This study lays a strong foundation for progressing the use of HPDGS inhibitors such as PK007, which specifically inhibit PGD2 and reduce inflammation, as a viable therapeutic approach for DMD. This approach protects dystrophic muscles from necrosis and reduces the severity of this debilitating disease, improving outcomes and quality of life.

**Supplementary Information:**

The online version contains supplementary material available at 10.1186/s13395-025-00379-1.

## Highlights

• Novel therapeutic approach: PK007 is a selective hematopoietic prostaglandin D2 synthase (HPGDS) inhibitor targeting Duchenne Muscular Dystrophy (DMD).

• Efficacy in *mdx* mice: PK007 significantly reduces PGD2 levels, myonecrosis, and macrophage infiltration in dystrophic muscles.

• Functional benefits: PK007 improves muscle strength and locomotor activity in juvenile *mdx* mice during acute disease progression.

• Clinical potential: PK007 demonstrates potential as a targeted therapy for DMD.

**Supplementary Information:**

The online version contains supplementary material available at 10.1186/s13395-025-00379-1.

## Introduction

Duchenne Muscular Dystrophy (DMD) is a severe, progressive X-linked disease characterised by chronic inflammation, muscle damage and necrosis, with consequent loss of muscle mass [13, 23, 47]. Early events of DMD (acute phase) include cycles of muscle damage resulting in myonecrosis associated with inflammation, with consequent myogenesis and muscle regeneration; however, progressive cycles of damage and inflammation increase fibrosis that impairs myogenesis leading to muscle replacement by fibrous fatty connective tissue [1, 75]. Consequently, DMD boys usually become wheelchair dependent around 10–12 years of age and require assisted ventilation by 20 years [38]. While life expectancy can now extend to about 40 years, premature mortality occurs predominantly due to cardiac and respiratory failure from progressive muscle weakness [23, 52].

DMD is caused by an inherited genetic defect in the dystrophin gene, preventing the production of the muscle isoform of dystrophin, Dp427m [23, 83]. Almost all muscles are affected, with the onset of the disease primarily affecting the hindlimbs, specifically the *gastrocnemius* (GA), *tibialis anterior* (TA) and the *extensor digitorum longus* (EDL) muscles [53]. The absence of dystrophin results in fragility and damage to the muscle membrane (sarcolemma), compromising the structural integrity of the myofiber and resulting in myonecrosis [10]. Initiation of myonecrosis triggers a complex inflammatory-immune response [79], with the production of cytokines (such as TNF-α, TGF-β, interleukin-6 and interleukin-1) and breakdown of the extracellular matrix that attracts neutrophils and macrophages to the site of damage [79]. These cells produce highly reactive oxidative species that exacerbate the myofiber damage, such as hypochlorous acid produced by neutrophils and macrophages [41]. The pro-inflammatory macrophages exacerbate the inflammatory process by promoting further muscle fibre lysis via the production of inducible nitric oxide synthase (iNOS) [43, 80]. Subsequently, this mechanism by immune cells amplifies muscle damage, highlighting the role of the immune system in the disease's progression [42, 44].

The classic clinical treatment for DMD is the use of glucocorticoids that have anti-inflammatory properties, such as Prednisone/Prednisolone/Deflazacort [58] and more newly available Valmorolone [27, 40]. Glucocorticoids mitigate inflammation by acting on the cyclooxygenase (COX) pathway, mainly by inhibiting the isoform COX-1 [40, 45, 58, 67]. However, long-term treatment with glucocorticoids is associated with adverse effects such as cushingoid features, obesity, growth retardation, hypertension, bone demineralisation, increased infection risk, peptic ulcers, and cardiovascular problems [86]. Moreover, Prednisone targets upstream of COX-1, it inhibits other beneficial, downstream prostaglandin targets that aid in muscle regeneration [45, 58]. Prostaglandins are homeostatic lipid mediators derived from arachidonic acid,the four prostaglandins (PGs) are PGD2, PGE2, PGF2α, and PGI2 (also known as Prostacyclin) [32]. The anti-inflammatory roles of PGE2, PGF2α and PGI2 have been reported in DMD patients, primarily aiding in muscle repair and regeneration [30, 32]. By contrast, PGD2 plays a pro-inflammatory role, amplifying chemokine and interleukin signalling and immune cell recruitment, further aggravating muscle damage [30, 32]. Thus, the selective targeting of PGD2 was the central aim of our study.

Due to the concerns about adverse problems with glucocorticoids for DMD, alternative novel, efficacious therapeutics to target inflammation, more specifically, are needed. The literature has explored the potential of inhibiting the production of PGD2 via hematopoietic prostaglandin D2 synthase (HPGDS) inhibitors [15, 54]. To date, oral HPGDS inhibitors, such as TAS-205, have been tested in DMD boys [39], whilst HQL-79 [54] and PK007 (our novel compound) have been tested in the *mdx* mouse model of DMD [84]. Unlike Prednisone, HPGDS inhibitors, like PK007, target the pro-inflammatory downstream PGD2 whilst preserving beneficial anti-inflammatory prostaglandins like PGE2, PGI2, and PGF2α [66]. The use of PK007 aims to decrease myonecrosis, the critical feature of DMD, without the broad immunosuppressive and adverse effects of glucocorticoids, offering a promising alternative treatment for DMD therapy.

This present study examines (i) pharmacokinetics to understand the novel characterisation of PK007 more fully, including in vitro potency and in vivo pharmacokinetic profiles: vital information essential for future potential clinical translation. We have analysed the impact of PK007 in *mdx* mice in more depth and extended our preliminary findings in this dystrophic mouse model [84]. The new analyses (ii) include the impact of PK007 on endogenous PGD2 levels and macrophage numbers in a range of muscles and on ambulation and oxygen consumption (VO_2_ levels) in *mdx* mice. Furthermore, these new data substantiate the benefits of PK007 on increased muscle strength and decreased myonecrosis at a higher confidence level of 90% with increased biological numbers. These studies substantiate the novel benefits of PK007 in juvenile *mdx* mice (at the acute stage of dystropathology) as a model for young DMD boys.

## Methods

### In vitro and in vivo assays to test the pharmacology of PK007

#### Fluorescence polarisation enzyme-based assay to test the HPGDS inhibitory activity (potency) of PK007

A Cayman Chemical (Ann Arbor, MI, USA) Prostaglandin D2 Synthase (hematopoietic-type) Fluorescence Polarization (FP)-Based Inhibitor Screening Assay Kit – Green (Catalog #600007), was used to determine the HPGDS inhibitory activity (potency) of PK007. This biochemical assay was performed in a 384-well plate format. The manufacturer’s instructions were followed. In brief, an assay cocktail containing HPGDS fluorescent probe green, HPGDS fluorescent probe glutathione, HPGDS (human recombinant), and fluorescent probe assay buffer concentrate (1X) was prepared. PK007 stock solution was made to 10 µM in DMSO and serially diluted to give 12 concentration points (10 - 0.005 µM). PK007’s potency was calculated by its IC50 value, seen through the displacement of the fluorescent probe from its active site, leading to decreased fluorescence polarisation. PK007 was tested in technical triplicates across 3 assays (*n* = 3). After a 60-min incubation in the dark on a plate shaker, a Tecan M1000 Pro Plate Reader (Tecan Group Ltd, Mannedorf, Switzerland) was utilized (470 nm excitation and 530 nm emission) at room temperature. Nonlinear regression analysis of log (concentration) versus normalised response determined the IC50 value of PK007 via GraphPad Prism software (version 10.2.2., Boston, MA, USA, www.graphpad.com). Outliers, if any, were identified and removed using the ROUT method (Q = 1%) [55]. Data displayed as mean ± SEM.

#### Macrophage cell culture and maintenance: for testing PK007

The murine macrophage line (RAW 264.7; American Tissue Culture Collection [ATCC] – In Vitro Technologies Vic, Australia) was maintained in a humidified environment of 5% CO_2_ at 37 °C. RAW 264.7 cells were cultured in RPMI1640 media (Gibco, ThermoFisher Scientific, Waltham, MA, USA) containing 5% fetal calf serum (FCS, heat-inactivated; Gibco, ThermoFisher Scientific) and 1% Glutamax (Gibco, ThermoFisher Scientific). RAW 264.7 macrophages were semi-adherent and kept at a confluency below 70%; media was renewed every 2 - 3 days. Cells were directly split from the plate (split 1:3 to 1:10 into fresh media) or harvested, washed, resuspended and plates seeded with completely fresh media. Both floating and adherent cells were harvested. Cells were rinsed once with phosphate-buffered saline pH 7.4 (PBS) and harvested with 0.25% Trypsin-EDTA. The remaining cells were cryopreserved in freezing media containing 90% FCS and 10% DMSO v/v. In the exponential phase, the double time of the macrophages was 18 h.

#### PGD2-MOX ELISA to assess the cell-based HPGDS inhibitory activity (potency) of PK007

The RAW 264.7 macrophages were seeded onto a 96-well plate at 50,000 cells/well density. PK007 was dissolved in DMSO and serially diluted (10 - 0.000169 µM) in a growth medium (as above), with DMSO concentration < 1% of the final volume and applied to the macrophages for 30 min. After incubation, the macrophages were activated by medium containing lipopolysaccharide (LPS) (500 ng/mL; *Salmonella typhosa* (Sigma-Aldrich, St. Louis, USA)) for 6 h. The supernatant was collected to quantify the PGD2 generated using a Prostaglandin D2-MOX ELISA kit (Cayman Chemical, Catalog #512,011). The manufacturer’s instructions were subsequently followed. Each concentration was assayed in technical duplicates or triplicates; the assay was conducted as *n* = 4. Absorbance was measured at 414 nm on a Tecan M1000 Pro Plate Reader (Tecan Group Ltd.). Nonlinear regression analysis was performed as stated previously.

#### PK007 treated, LPS-stimulated macrophages were evaluated for cell survivability by 3-[4,5-dimethylthiazol-2-yl]−2,5 diphenyl tetrazolium bromide (MTT) assays

The MTT assay is a quantitative and sensitive detection of cell survival, measured through metabolic processes; the reduction of a colourimetric substrate by mitochondrial enzymes was measured [72]. RAW 264.7 macrophages were seeded at 50,000 cells/well in a growth medium (as above), treated for 30 min with PK007 (100 - 1 µM) and then stimulated with LPS (500 ng/mL) for 6 h. The growth medium was replaced with 1 mg/mL MTT (dissolved in growth medium) (Sigma Aldrich) and incubated at 37 °C for 1 h; in healthy cells, the MTT yellow tetrazolium dye can be converted by mitochondrial enzymes into an insoluble purple formazan product. After incubation, the MTT solution was removed, and the tetrazolium salt was dissolved with isopropanol for 10 - 15 min; the plate was read at 570 nm on a Tecan M1000 Plate Reader (Tecan Group Ltd.). Data exhibited as mean ± SEM.

#### Pharmacokinetics of PK007 plasma levelsin vivo

Pharmacokinetics (PK) of PK007 were assessed by WuXi AppTec Co., Ltd. (Shanghai, China; hereafter referred to as WuXi AppTec). This study was conducted with animal ethics approval: the Animal Care and Use Programs at WuXi AppTec have been fully accredited by the Association for Assessment and Accreditation of Laboratory Animal Care (AAALAC) and certified by local municipal or provincial Commission on Science and Technology (Shanghai, China). This study was in accordance with the WuXi AppTec Institutional Animal Care and Use Committee (IACUC) standard animal procedures, along with the IACUC guidelines (IACUC Number: PK01-SH004-2023v1.0) that follow the Australian Code for the Care and Use of Animals for Scientific Purposes (Australian National Health and Medical Research Council 8th edition, ISBN:1,864,965,975).

WuXi AppTec administered PK007 to classic male C57BL/6 mice, as this strain offers a well-characterized baseline for evaluating pharmacokinetics and drug metabolism [9]. In brief, PK007 was administered as a bolus dose intravenously (IV, *n* = 3) and orally (PO via gavage, *n* = 3). IV vehicle formulation was 0.4 mg/mL in PEG400: water (70:30), whilst PO vehicle formulation was 1 mg/mL in PEG400: water (70:30), achieving a clear solution for both. The target dose was 2 mg/kg and 10 mg/kg for IV and PO doses, respectively. The concentration of PK007 in blood plasma was sampled at 8-time points: IV (0.083, 0.25, 0.5, 1, 2, 4, 8, and 24 h) and PO (0.25, 0.5, 1, 2, 4, 6, 8, and 24 h) Phoenix WinNonlin 6.3 software (Certara, Radnor, PA, USA) was utilised to calculate any PK parameters and data were fitted to a non-compartmental model; the calculation method was linear/log trapezoidal. Data presented as mean ± SEM. 

### Animal studies

#### Ethics statement

All animal work at the University of Queensland was approved by the University of Queensland Animal Ethics Committee (Ethics Number: AE000047) and complied with the policies and regulations regarding animal experimentation. Studies were conducted per the Queensland Government Animal Research Act 2001, associated Animal Care and Protection Regulations (2002 and 2008) and the Australian Code of Practice for the Care and Use of Animals for Scientific Purposes, 8th Edition (National Health and Medical Research Council, 2013; ISBN:1,864,965,975). ARRIVE guidelines have been followed in the preparation of the manuscript [35, 51].

#### Drug study design

Three-week-old male dystrophic C75BL/10 ScSn-*mdx (mdx*) mice and C75BL/10 ScSn, the parental strain control (termed wild type (WT)) mice, were used in this study, sourced from the Animal Resources Centre (ARC) Perth, WA, Australia. These mice were obtained by ARC under licence from the Jackson laboratory (jax.org) (*mdx* Jax stock number 0001801 and WT Jax stock number 000476) and represent the most used and best-characterized mouse model of DMD (see JAX laboratory *mdx* physiology data sheets jax.org). Standard pre-clinal treatment guidelines for *mdx* and WT mice were used throughout this study [17]. As previously described, mice were randomly allocated into two groups in a double-blind manner. This allocation was performed by animal house technicians and not the experimenter. They were housed individually in ventilated or metabolic cages for PhenoMaster studies (see below) and fed a standard meat-free mouse diet (Speciality Feeds, W.A. Australia, Cat Number SF00-100) that meet National Research Council USA standards (10.17226/4758). Mice were treated in a double-blind manner with vehicle (0.5% methylcellulose, 0.1% Tween80, and MilliQ water) or the drug PK007 (10 mg/kg/day in 0.5% methylcellulose, 0.1% Tween80, and MilliQ water) via oral gavage daily at 9.30 am and administered for 10 days, from postnatal day 18 to 28 days [84]. In brief, our double-blinded design involved coding PK007 and vehicle by a third party (i.e., non-lab member) and coding the genotype of *mdx* and WT (strain control) mice by animal house technicians. This process allowed the genotype and treatment to be hidden from the experimenter until each experiment was completed. This included data collection and analyses. Supplementary Figs. 1 and 2 schematically summarises our drug study design, which accords with standard operating procedures provided by the Treat NMD international study group (See SOPs Webpage [73]). These procedures also conform to ARRIVE guidelines [51] .

#### Muscle grip strength: assessment of function

Hindlimb grip strength was measured as described previously [84]. PK007, Vehicle, Prednisone and WT-treated mice were measured daily, two hours after oral gavage over the 10-day treatment period and assessed using the IMADA® grip device (UGO Basile, Cat#47,200). The instrument measured the highest force generated by each mouse throughout five trials during a 1-min cycle. The measurement was measured in gram force and then converted to newtons. This methodology followed TREAT-DMD standard operating procedures that have been published for the pre-clinical assessment of drugs (standard operating procedure (SOP ID: DMD_M.2.2.001 [47]).

#### PhenoMaster: assessment of behaviour

A new cohort of *mdx* and WT mice were individually housed in the ‘PhenoMaster’ (TSE systems, Germany) to characterise each mouse’s metabolic and real-time live movement patterns (using 10 individual cages simultaneously simultaneously). Specifically, its software can assess the frequency of food and water intake; indirect calorimetry measurements of animal oxygen consumption; carbon dioxide production to calculate key metabolic parameters (respiratory exchange ratio); and ActiMot light sensors to monitor movements (in an x, y, z plane; see Supplementary Fig. 2). We continuously monitored each mouse for total activity and VO_2_ over 24 h, recording data hourly: this analysis was done continuously for 10 days. To investigate variations in activity levels, we divided the data into light (6 am-6 pm) and dark (6 pm-6 am) cycles, analysing differences during the most metabolically active period of mice, the dark (nocturnal) cycle.

#### PGD2 MOX-ELISA – to quantify mdx serum PGD2 levels

Blood was collected via cardiac bleed (at 28 days), placed into cryovials, centrifuged at 4000 rpm for 10 min at 4 °C, and serum was collected and used fresh. A PGD2-MOX ELISA kit was used to determine PGD2 levels (Cayman Chemical, Catalog# 512,011). Samples were run in duplicate and were measured at OD 450 nm on a Tecan M1000 Pro Plate. A standard curve was generated, and a sigmoidal 4PL curve was used to calculate unknown concentrations per the kit’s instructions. Analysis was performed in GraphPad Prism (California, USA, version 10.2.2).

#### Muscle tissue sections and histology

Muscles from the hindlimb (GA, TA, and EDL), the diaphragm, and the tongue were dissected from mice at the end of the treatment period (at 28 days). These muscles were cut at the mid-point between origin and insertion to ensure that the same muscle region was analysed across genotype and treatment groups. Samples were then fixed in 4% paraformaldehyde–phosphate-buffered saline pH 7.4 (PBS) overnight at 4 °C, washed in PBS, dehydrated (70% ethanol, 90% ethanol, 100% ethanol (2 ×), and xylene (3 ×)), and processed into paraffin blocks. Transverse sections were cut at 7 µm using a Lecia RM 2245 (Nußloch, Germany) microtome and collected onto Super Frost Plus microscopic slides. These slides were then stained with Mayer’s hematoxylin and eosin (H&E) for characterisation of myonecrosis [26], and with picrosirius red stain for quantification of fibrosis which stains type I collagen fibres red and type III collagen fibres yellow. Fibrosis was quantified as the percentage of collagen-stained area relative to the total muscle cross-sectional area. Muscle sections were scanned digitally using the ZEISS Axioscan microscope slide scanner at 20 × objective (a numerical aperture of 0.4). Myonecrotic areas were quantified using the open software “QuPath” version 0.4.3 (https://qupath.github.io). The total area of myonecrosis was calculated by the program for each mid-sectional transverse section of the sample (i.e., mid-point along the longitudinal axis of the muscle origin to insertion [8, 84]). The total area of the sample was then calculated to determine the total percentage of necrosis relative to each sample size. GraphPad Prism (version 10.2.2) was used to determine statistics and construct graphs.

#### Immunofluorescence to quantify macrophages in muscles

Immunostaining to identify macrophages (using anti-Iba1) was performed on hindlimb muscles (GA, TA, and EDL) in fixed paraffin sections (*as described above*). In brief, sections were rehydrated (xylene × 3, ethanol × 2, 90% ethanol, 70% ethanol, running distilled water for 2 min each), and antigen retrieval was performed using sodium citrate buffer (10 mM sodium citrate, 0.05% Tween 20, pH 6.0) for 10 min using a microwave (900-W output). Sections were blocked in 0.1% Triton X-100 in PBS (T-PBS) containing 5% normal donkey serum (Sigma-Aldrich, Missouri, USA) for 1 h [14]. Sections were then incubated with either polyclonal antibody anti-Iba1 (ab5076, goat polyclonal; Abcam Inc., MA, USA) diluted 1:500 in blocking buffer at RT for 18 h, anti-iNOS (ab3523, rabbit polyclonal; Abcam Inc., MA, USA), anti-IL-1β (P420B, rabbit polyclonal; Thermo Fisher Scientific, MA, USA), or anti-TNF-α (#MM350C, mouse monoclonal; Thermo Fisher Scientific, MA, USA). After incubation, sections were washed (3 × 10 min) with T-PBS. Sections were then incubated in secondary polyclonal antibody Alexa-647 anti-goat (705–607-003, donkey polyclonal; Jackson ImmunoResearch, PA, USA) diluted 1:500 in blocking buffer at RT for 2 h, followed by 3 × 10 min wash with T-PBS. Staining procedures were standardised between sections. Sections with no primary antibodies were stained with secondary antibodies as negative controls for non-specific background staining.

The muscle sections were imaged using the Leica DMi8 inverted widefield microscope equipped with Spectra X LED excitation lines (390 and 513 nm), running Leica LAS X software (Leica Microsystems, Wetzla, Germany). Images were taken at a resolution of 1024 × 1024 pixels using a 20 × air objective (NA 0.7). Laser power levels and exposure times were maintained at the same level across all tissue sections between the PK007 and Vehicle-treated *mdx* mice. All images were reconstructed and analysed using Fiji imaging software [63]. The images were then saved in TIFF format.

### Experimental design, statistical analyses and animal usage

Drug identity and post-experimental analyses were conducted double-blinded for all animal experiments, as detailed above. This also included the random assignment of *mdx* and WT mice to either treatment group (PK007 treatment or Vehicle). As noted in each figure legend, statistical analyses were performed on animal studies with a stated *n* value (between *n* = 6/treatment to *n* = 12/treatment). All data were assessed for distribution using Shapiro–Wilk normality tests. Data that passed this test were analysed using a two-way ANOVA with Tukey’s multiple comparison test to assess animal strain differences. For comparisons between PK007-treated and vehicle-treated *mdx* mice, an unpaired two-tailed *t*-test was used. All statistical analyses were performed with GraphPad Prism 10.2.2 software (GraphPad Software, Inc., California, USA). Sample sizes were subjected to statistical analysis, where *n* = number of independent values. All values are presented as mean ± standard error of the mean (SEM). Significance was set at *p* < 0.05 for all tests.

## Results

### Pharmacology: PK007 displays nanomolar in vitro potency and half-life in vivo

**Fig. 1:**
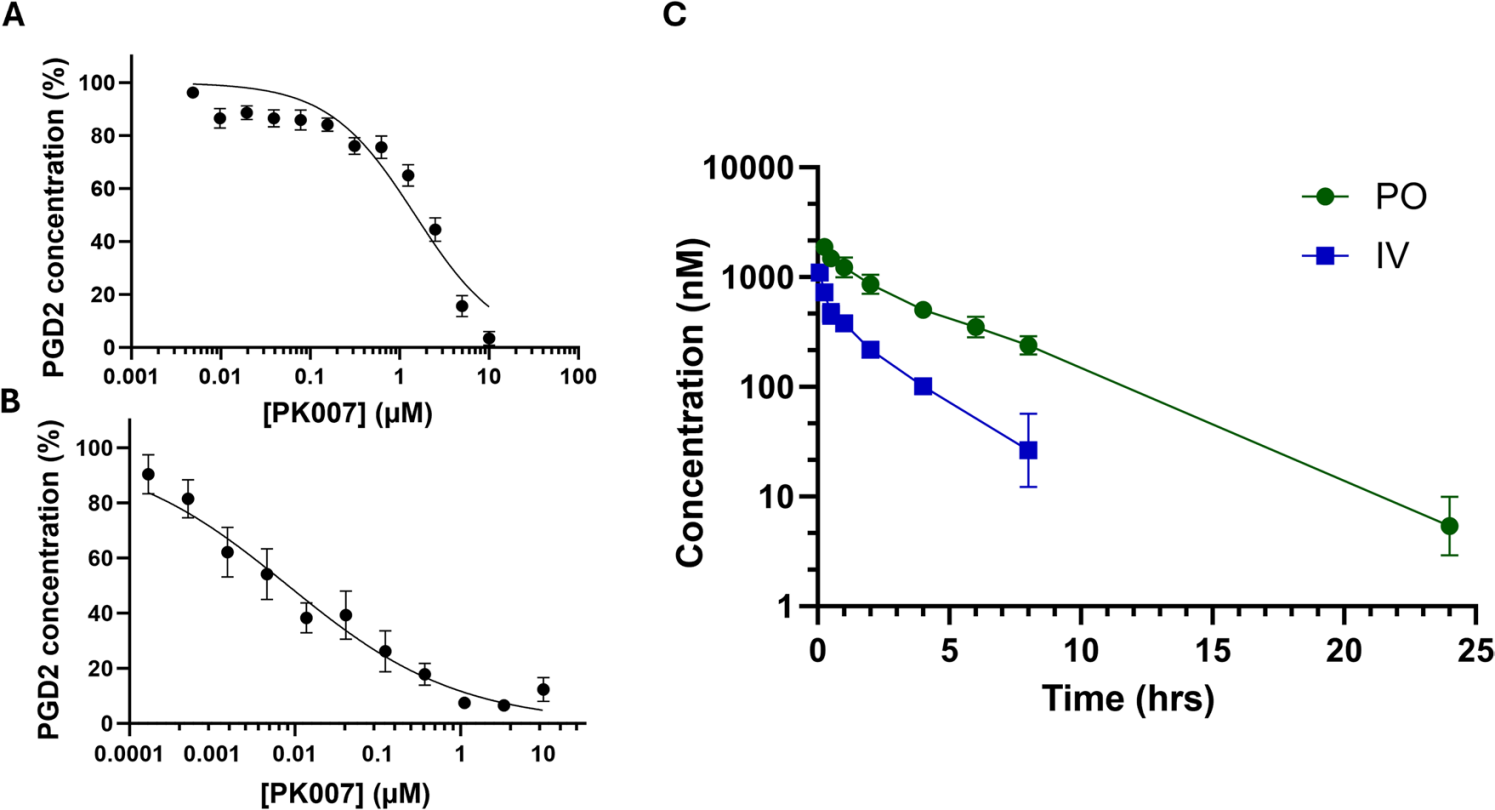
In vitro studies demonstrated nanomolar potency and showed excellent pharmacokinetics of PK007. **A** displays the inhibitory activity of PK007 on PGD2 concentration (%) via fluorescence polarisation (FP) enzyme-based assays, where potency was 1.54 ± 0.26 µM. The assay was conducted with triplicate replicates, with over three assays (*n* = 3). **B** The cell-based potency of PK007, when measured via PGD2-MOX ELISA in LPS-stimulated macrophages, showed that the inhibition of PGD2 was 17.23 ± 12 nM. The assay was conducted in technical duplicates or triplicates over four assays (*n* = 4). GraphPad Prism software utilised a non-linear regression algorithm for log (concentration) versus normalised response. **C** Pharmacokinetics to determine the in vivo half-life of PK007 in blood plasma was determined in C57BL/6 mice, using two different delivery regimens (*n* = 3 for each treatment), orally (PO, 10 mg/kg) and intravenously (IV, 2 mg/kg). The sampling time was over 24 h (hrs), and plasma concentration was measured (nM). Pharmacokinetic evaluations were conducted by WuXi AppTec (Shanghai, China). Data are shown as mean ± SEM

These new data describe the potency and pharmacokinetics of PK007. Potency was determined using two different models: a biochemical fluorescence polarisation (FP) enzyme-based assay (Fig. 1A) and the PGD2-MOX ELISA cell assay using a macrophage cell line (Fig. 1B). The FP assay showed the potency was 1.54 ± 0.26 µM (Fig. 1A), whereas nanomolar concentration was achieved when quantified in the PGD2-MOX ELISA cell-based assay (17.23 ± 12 nM; Fig. 1B). The impact of PK007 on cell survival was also tested in an MTT assay; cells treated with PK007 for 6.5 h demonstrated excellent cell survivability (> 85%) at concentrations 30—1 µM (Supplementary Fig. 3).

For subsequent in vivo studies, it was imperative to understand the pharmacokinetics of PK007. Specifically, we sought to determine its bioavailability and metabolic stability in vivo, largely determined by the general physiology and hepatic metabolic pathways, independent of mouse strain. PK007 was administered to C57BL/6 mice (*n* = 3) both orally (PO, 10 mg/kg) and intravenously (IV, 2 mg/kg); the plasma concentration was determined over time. Following a single IV administration of PK007, the mice showed low plasma clearance (50.4 ± 4.8 mL/min/kg) and high volume of distribution (VD_ss_) (7.3 ± 4.6 L/kg), with a half-life of 1.9 ± 0.3 h. Immediately after injection (C0), the concentration was 259 ± 10.1 nM (Fig. 1C). After the oral bolus dose, peak plasma concentration was observed at 0.25 h, suggesting rapid absorption; C_max_ was 356 ± 16.4 nM. PK007 displayed a long oral half-life of 3.0 ± 0.3 h (Fig. 1C). The bioavailability of the compound was found to be 81%. Refer to Supplementary Table 1 for the data overview. In summary, PK007 exhibited low clearance, high volume of distribution, long half-life, and good oral bioavailability in C57BL/6 mice.

### Grip strength in PK007-treated mdx mice is significantly increased, and MOX-PGD2 levels decreased

**Fig. 2:**
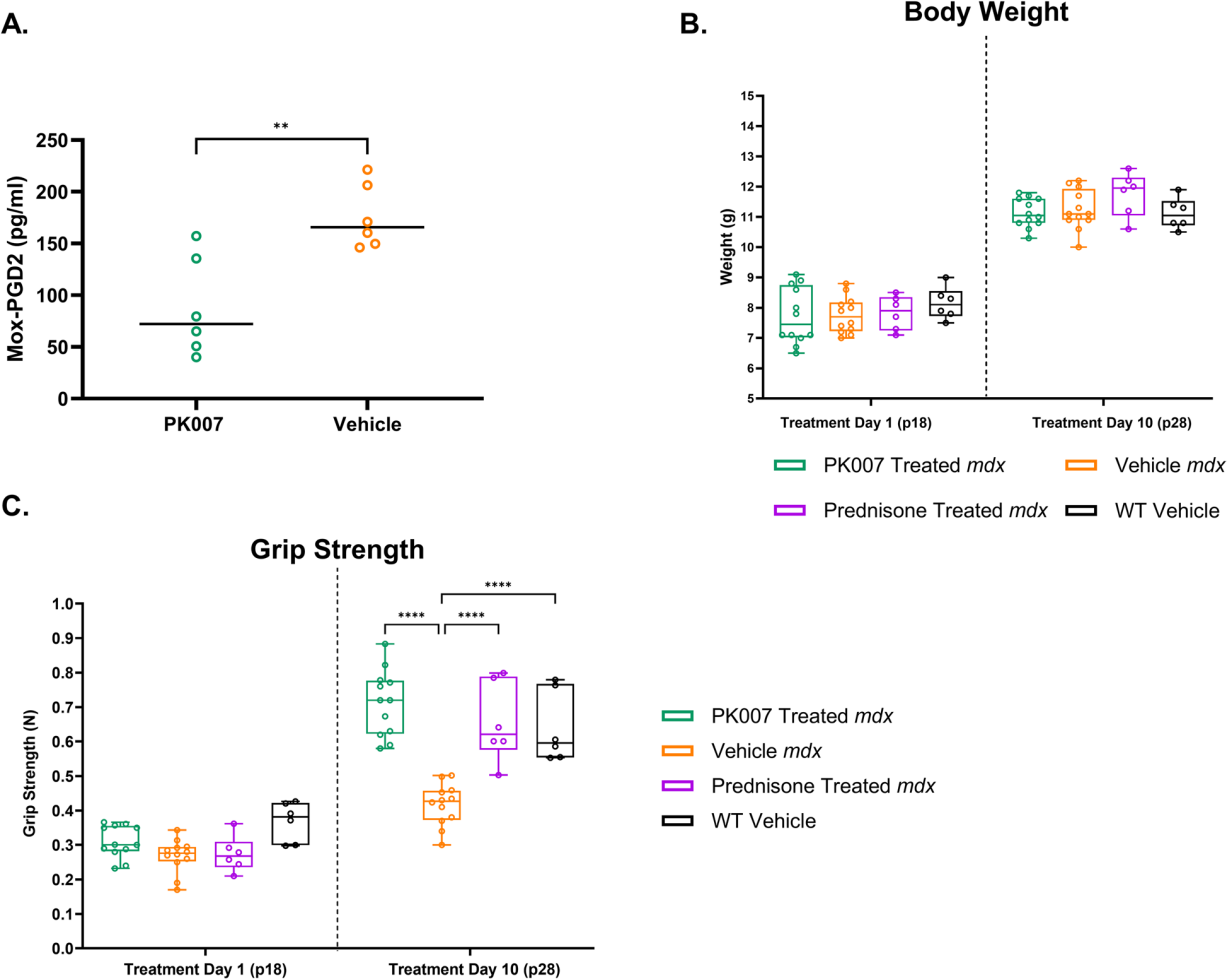
PK007-treated mice display lower PGD2 levels and increased grip strength comparable to Prednisone-treated *mdx* and WT mice. **A** PK007-treated mice display a significant decrease in PGD2-MOX concentration (pg/ml) compared to vehicle-treated *mdx* mice. The mean ± SEM values of blood PGD2 concentration levels are shown. An unpaired two-tailed *t*-test calculated biological *n* = 6 and *p*-values. **p = 0.036. **B** displays no significant change in mean body weight over the day 1 (postnatal day 18 [p18]) and day 10 (postnatal day 28 [p28]) treatment period for PK007 treated (green), Vehicle *mdx* (orange), Prednisone-treated *mdx* (purple) and Vehicle WT (black). **C** denotes a significant increase in grip strength (compared with Vehicle-*mdx*) for PK007 and Prednisone-treated *mdx* mice and WT mice on day 10. Biological *n* = *12* for Vehicle and PK007 *mdx* groups. Biological *n* = *6* for Prednisone-*mdx* and WT control mice. *P* values were calculated using a two-way ANOVA with Tukey’s post hoc test. **** denotes *p* < 0.0001

To measure PGD2 levels in the serum of *mdx* mice sampled at 28 days of age, we employed a MOX-PGD2 ELISA kit to convert unstable PGD2 to stable MOX-PGD2. Figure 2A displays a significant (*p = 0.036; *n* = 6) reduction of serum PGD2 in the PK007-treated *mdx* mice (mean concentration of 88.02 pg/ml) compared to the vehicle-treated mice (mean concentration of 175.85 pg/ml) *mdx* mice. This result indicates that PK007 effectively blocked the production of PGD2 via HPGDS; subsequently, we wanted to assess if this had any therapeutic benefit in the drug-treated cohort.

Having established that PK007 effectively reduces PGD2 levels in treated *mdx* mice, we next evaluated body weight and grip strength as measures of muscle strength. This grip strength analysis builds on our previous findings [84] by using a larger sample size (*n* = 12, compared to *n* = 6) and incorporating comparative data with Prednisone-treated *mdx* mice and wild-type controls. Throughout the 10-day treatment period, the body weight of the juvenile mice did not significantly vary amongst PK007, Prednisone (used only for grip and body weight assessments), and Control-treated mice (Fig. 2B). On treatment day 1 (postnatal day 18), grip strength did not differ significantly across all groups (Fig. 2C, left). However, by day 10 (postnatal day 28), grip strength significantly increased in the PK007 (0.71N ± 0.03) and Prednisone (0.66N ± 0.05) treated *mdx* mice, as well as the strain control (0.64N ± 0.04) mice compared to Vehicle-treated *mdx* mice (0.42N ± 0.02) (*p* < 0.0001, *n* = 6–12 in Fig. 2C). While there was no significant difference in muscle strength between PK007 and Prednisone-treated groups, PK007-treated *mdx* mice showed, on average, higher force output. These results suggest that reducing PGD2 levels increased hindlimb muscle strength, with force output comparable to in-market approved glucocorticoid Prednisone treated and strain control mice.

### Movement activity is increased in PK007-treated mdx mice

**Fig. 3:**
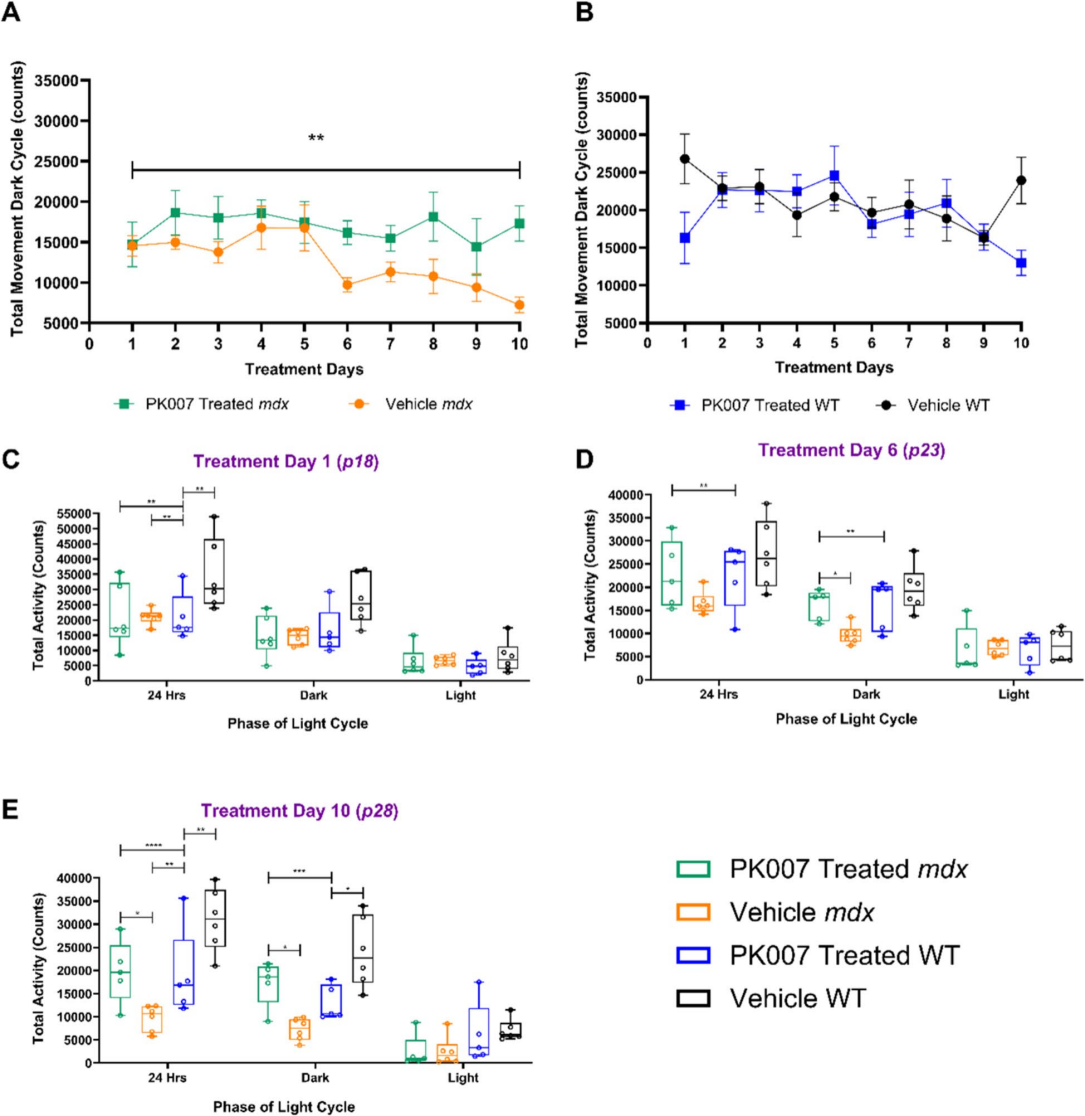
Increased movement activity in PK007-treated *mdx* mice. **A** highlights a significant increase in locomotor movement in the dark cycle between PK007-treated and Vehicle *mdx* mice over the 10-day treatment period (postnatal 18 to 28 days). ** denotes *p* value = 0.0014. Error bars represent the standard error of the mean, and an unpaired *t*-test was used to calculate the *p*-value. **B** represents no significant difference in locomotor movement between PK007-treated and Vehicle WT mice in the dark cycle over the 10-day treatment period (postnatal 18 to 28 days) *p-value* = 0.2735. Error bars represent the standard error of the mean, and an unpaired *t*-test was used to calculate the *p*-value. **C-E** denote the analysis of total movement activity over days 1, 6 and 10 across the four different treatment groups. Total movement activity was analysed at 24 h, the dark cycle (6 pm-6 am), where mice are the most active and the light cycle (6 am-6 pm), where the mice are least active. **C**, *p* values denote * = 0.0474, ** = 0.0090. **D**, * = *p* < 0.05. **E**, *p* values denote as follows: ** = 0.0010, *** = 0.0003 and **** = *p* < 0.0001. Biological *n* numbers = 6 between all treatment groups. *P*-values for panel B were calculated using a 2-way ANOVA with Tukey’s post hoc test

In addition to measuring grip strength, we compared the movement activity in groups of PK007-treated *mdx* mice and PK007-treated WT mice, control vehicle-treated *mdx* and WT mice. We used the PhenoMaster to track movement and VO_2_ levels over 24 h for each mouse for 10 days (postnatal days (PN) 18 to 28 [PN18 to PN28]). In general, we observed no change in movement activity over a 24 h period (total sum of movement) between PK007 and Vehicle-treated *mdx* mice. However, when locomotion was analysed at the period where mice were most active, dark cycle (6 am–6 pm), Vehicle *mdx* mice displayed significantly reduced activity. This is evident in Fig. 3A, where no significant difference in movement was observed in the first five days of treatment for *mdx* mice; on day 6, the control vehicle-treated *mdx* mice (orange data) significantly reduced activity compared to the PK007-treated *mdx* mice (green data). Further, Fig. 3B shows no difference in total movement activity in the dark cycle between PK007 treatment and WT mice.

Data for the dark cycle, which is the nocturnal, active phase for mice [3, 7] is further shown for 3 different days at 1, 6 and 10 (Figs. 3C, D and E). Figure 3C highlights no significant difference in movement activity on day 1 for any of the groups in the dark cycle. Figure 3D shows that on day 6, untreated (Vehicle) *mdx* mice decreased activity, whereas PK007-treated *mdx* mice maintained high activity, similar to the WT mice (PK007 and Vehicle-treated). Figure 3E shows that this decrease was more pronounced at day 10 for the untreated (Vehicle) *mdx* mice; this loss of function likely results from increased ongoing myonecrosis (manifesting by loss of function) due to exercise-induced damage to *mdx* muscles. It is impressive that at day 10, the higher activity (about double) was still maintained in the PK007-treated *mdx* mice, compared with the Vehicle-control *mdx* mice. These important data emphasise that PK007 protected muscle function during sustained locomotor activity, suggesting improved muscle function in the treated *mdx* mice over this 10-day period.

**Fig. 4:**
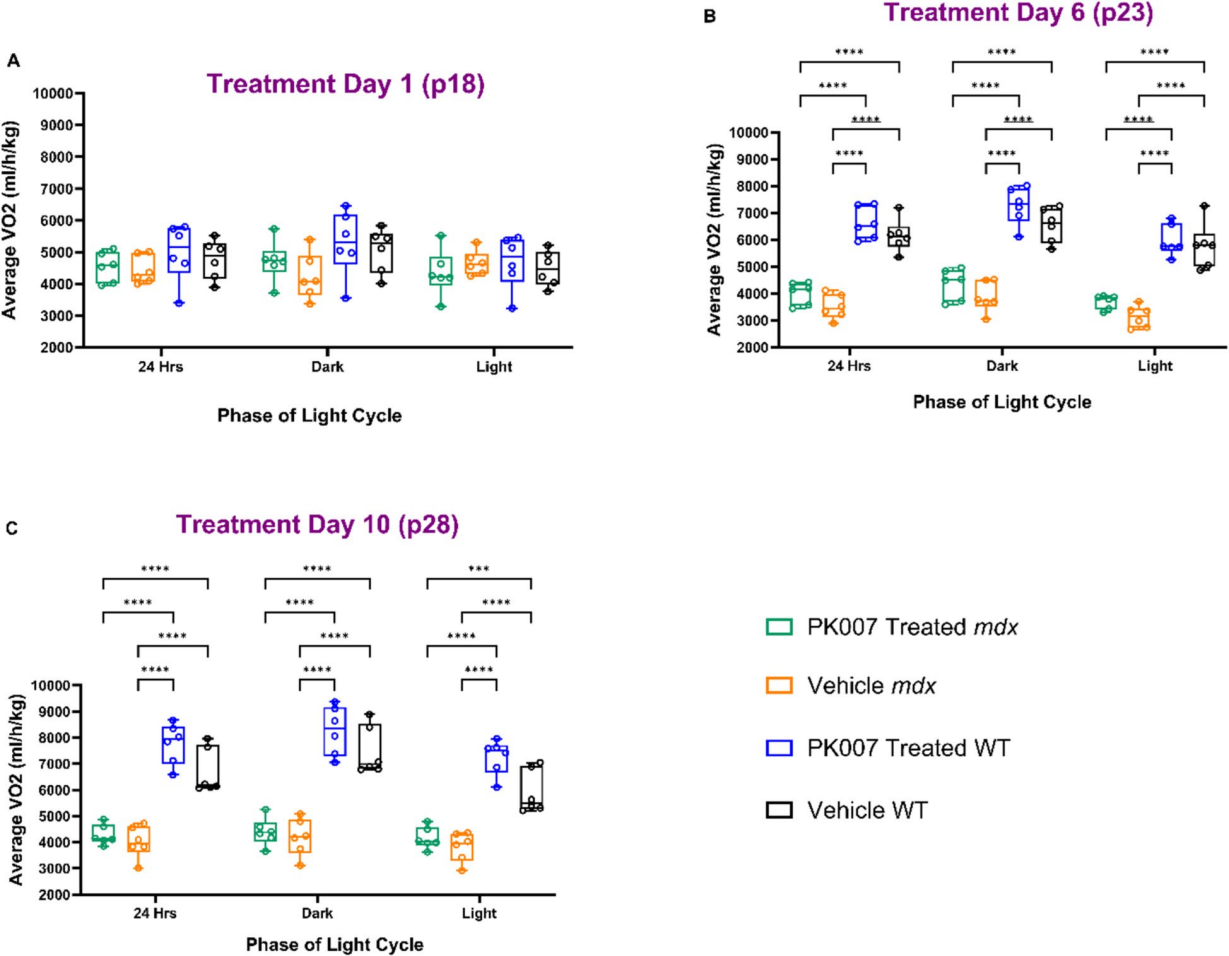
No significant change in average VO_2_ levels in PK007 and Vehicle-treated *mdx* mice. **A**-**C** denote VO_2_ levels of total movement activity over Days 1, 6 and 10 across the four treatment groups (Vehicle & PK007 treated *mdx* and WT groups). VO_2_ was analysed across 24 h and presented separately for the dark and light cycles. **A** displays no significant increase in VO_2_ levels at day 1 of treatment in a 24-h, dark and light cycle. **B** represents a significant difference between *mdx* and WT groups in VO_2_ levels in 24 h, dark and light cycle at treatment day 6. *P* values are denoted as follows: **** = *p* < 0.0001. **C** denotes a significant difference between *mdx* and WT groups in VO_2_ levels in 24 h, dark and light cycle at treatment day 10. *P* values are *** = 0.004 and **** = < 0.0001. Biological *n* = 6 between all treatment groups. *P*-values were calculated using a 2-way ANOVA with Tukey’s post hoc test

In parallel, we utilised the PhenoMaster to assess VO_2_ levels, a readout for oxygen consumption throughout the treatment period (10 days [p18 to p28]) for the 4 groups of PK007 and Vehicle-treated *mdx* and WT mice. These data for VO_2_ are presented for the entire 24 h and the dark and light phases for days 1, 6 and 10 (Fig. 4). On day 1, all groups showed no significant difference in VO_2_ levels (Fig. 4A). Most notably, from day 6, there was no significant difference in VO_2_ levels between PK007 and Vehicle-treated *mdx* mice (Fig. 4B). However, both groups of *mdx* mice displayed significantly less VO_2_ levels (mean levels of 3990.71 ± 201.54 ml/h/kg, 3504.08 ± 186.89 ml/h/kg respectively for PK007 and vehicle-treated) compared to the WT mice, treated with PK007 or Vehicle (mean levels of 6220.25 ± 621.16 ml/h/kg and 5821.86 ± 456.74 ml/h/kg respectively). This difference between *mdx* and WT mice was maintained until the end of the treatment period (day 10, [p28]; Fig. 4C). This result highlights that VO_2_ levels were low in these juvenile *mdx* mice. Similar VO_2_ levels were observed in the dark cycle (active phase) to the light phase (resting period). This could denote impaired respiration in the acute growth stage where energy demands are high [59], while PK007 did not improve VO_2_ levels; this may be due to the very high metabolic demands in the dystrophic muscles of these young *mdx* mice.

### Myonecrosis significantly decreased in PK007-treated mdx muscles

**Fig. 5:**
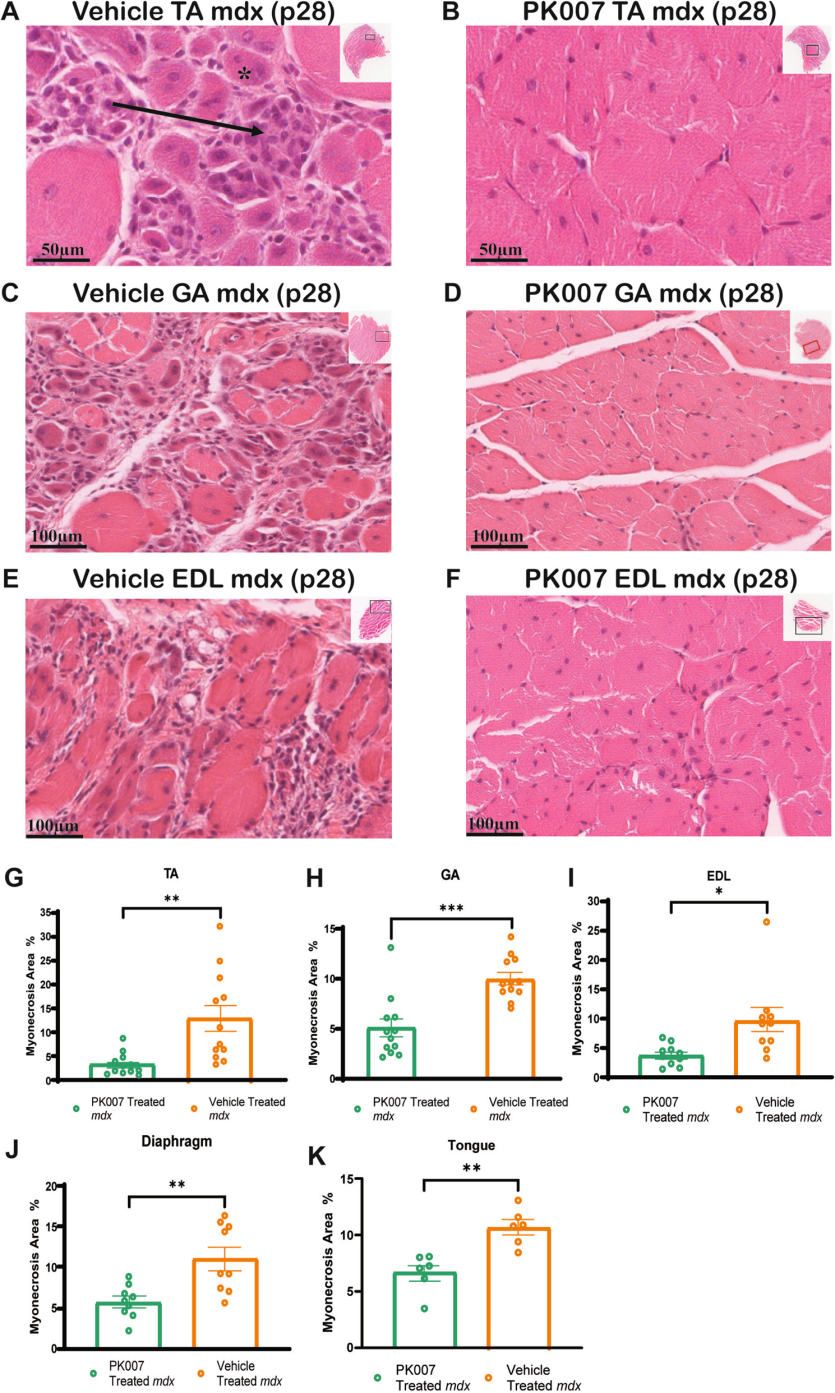
Myonecrosis in PK007-treated *mdx* muscles is significantly decreased after 10 days of treatment. Representative transverse H&E-stained sections of *tibialis anterior* (TA), *gastrocnemius* (GA), and *extensor digitorum longus* (EDL) muscles from vehicle-treated (**A**, **C**, **E**) and PK007-treated (**B**, **D**, **F**) *mdx* mice at postnatal day 28 (p28). Black arrows in vehicle-treated muscles highlight areas of necrotic myofibers characterized by pale cytoplasm and infiltrating immune cells, while asterisks (*) indicate regenerating myofibers with centrally located nuclei. Such features are markedly reduced in PK007-treated muscles. Insets in panels show the full muscle cross-sections. Quantification of myonecrosis (**G-I**) shows the percentage of necrotic area relative to the total muscle cross-sectional area in TA (**G**), GA (**H**), and EDL (**I**) muscles, demonstrating significant reductions in PK007-treated *mdx* mice compared to Vehicle-treated controls (*p = 0.0004, **p = 0.0165 and *** *p* = 0.0018). Panels **J** and **K** display similar analyses for the diaphragm (**J**) and tongue (**K**) muscles, where PK007 treatment also significantly reduces the myonecrotic area (diaphragm *p* = 0.0001 and tongue *p* = 0.0087). Data are presented as mean ± SEM, with individual points representing biological replicates, *n* = 12 for TA and GA, *n* = 10 for EDL, *n* = 9 for diaphragm, and n = 6 for tongue muscles. Statistical analyses were conducted using unpaired two-tailed *t*-tests

Myonecrosis in *mdx*/DMD is a result of the vulnerable sarcolemma and susceptibility to exercise-induced damage, with persistent inflammation: myonecrosis increases inflammation and results in myogenesis and muscle regeneration, as well as fibrosis over time [28, 46]. To extend our earlier observation suggesting reduced myonecrosis in PK007-treated *mdx* mice [84], we specifically quantified the areas of myonecrosis in the TA, GA, and EDL muscles (at p28 days) with an increased number of mice examined, biological, *n* = 12. Figures 5A-5F show the histological characteristics of myonecrosis (characterised by the compromised sarcolemma and clumped nuclei) pronounced in TA, GA and EDL muscles in untreated (vehicle) *mdx* mice compared with little myonecrosis in PK007-treated *mdx* mice at p28 days. These observations were quantified across the GA, TA and EDL muscles to show an approximate 50% decrease in myonecrotic area in PK007-treated *mdx* muscles compared to vehicle-treated *mdx* muscles (Fig. 5G-I). Statistical comparisons (mean ± SEM) and *p* values calculated using an unpaired two-tailed *t*-test) for PK007 and Vehicle-treated mice for the 3 muscles are shown here: GA 5.03% ± 0.90, vs 10.01% ± 0.62 [*p* = 0.0018] (Fig. 5G); TA 3.40% ± 0.66, vs 13.01% ± 2.70 [*p* = 0.0165] (Fig. 5H); and for EDL 3.85 ± 0.57 vs 9.70% ± 2.04, (*p* = 0.0004] (Fig. 5I). The significant decrease in myonecrosis in all these hindlimb muscles in PK007-treated mice strongly supports the proposal that decreasing PGD2 significantly protects these dystrophic muscles from myonecrosis.

We aimed to further evaluate myonecrosis in the diaphragm and tongue muscles, notably affected in DMD [36, 82]. These observations were quantified across the diaphragm and tongue muscles to show an approximate 40% decrease in myonecrotic area in PK007-treated *mdx* muscles compared to Vehicle-treated *mdx* muscles (Diaphragm 5.77% ± 0.68, vs 11.10% ± 1.40 [*p* = *0.0087]* (Fig. 5J; Supplementary Fig. 5A-5B); Tongue 6.66% ± 0.70, vs 10.73% ± 0.67 [*p* = *0.001]* (Fig. 5K; Supplementary Fig. 5C-5D). These results indicate that while VO_2_ levels were unaffected in PK007-treated mice, the reduced myonecrosis in the diaphragm and tongue muscles also accords with the benefits in the limb muscles.

### Fibrosis is minimal during the acute phase of DMD in mdx mice

Fibrosis is defined as the excessive deposition of extracellular matrix proteins, particularly collagen, within tissues, often resulting from chronic inflammation and tissue damage [34, 37]. In Duchenne muscular dystrophy (DMD), fibrosis is typically associated with the chronic phase of disease progression and is less pronounced during the early, acute phase characterized by myonecrosis and inflammation [37].

**Fig. 6:**
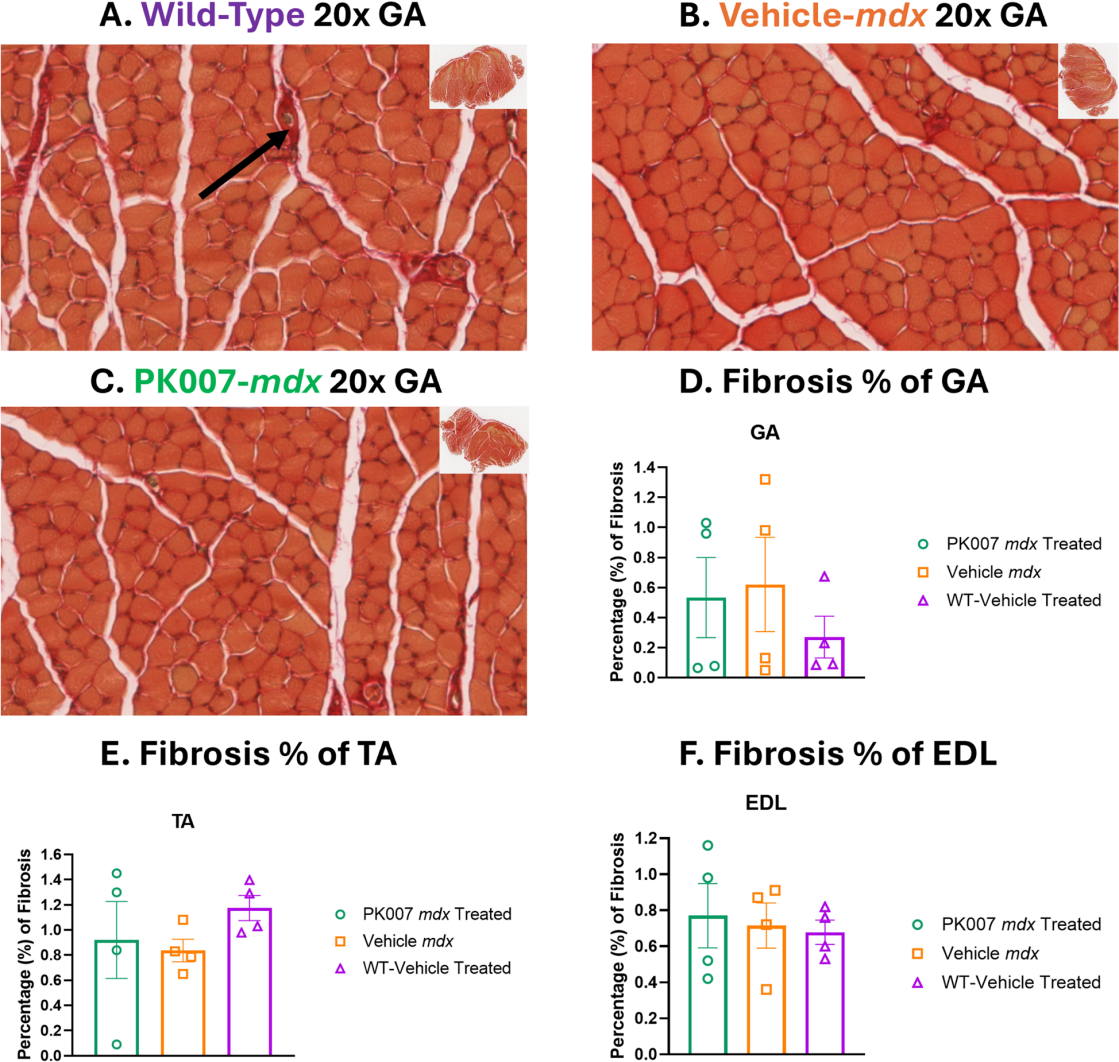
No significant change in fibrosis in wild-type and *mdx* muscles with Picrosirius Red staining. Representative transverse sections of hindlimb muscles stained with Picrosirius Red, showing collagen deposition in (**A**) wild-type (WT, purple), (**B**) vehicle-treated *mdx* (orange), and (**C**) PK007-treated *mdx* mice (green). Red fibres indicate mature type I collagen, while the black arrow highlights an area of collagen deposition, i.e., fibrosis. Insets display the full muscle cross-sections. Quantification of fibrosis as a percentage of total muscle area shows no significant differences in fibrosis percentages in the GA (**D**), TA (**E**), and EDL (**F**) muscles across PK007-treated, Vehicle-treated *mdx*, and WT mice. Data are presented as mean ± SEM, with individual points representing biological replicates (*n* = 4). Statistical analysis was performed using one-way ANOVA with Tukey’s post hoc test (*p* > 0.05)

To assess the presence of fibrosis during the acute phase of DMD, we performed Picrosirius Red staining on hindlimb muscles (GA, TA, and EDL) of *mdx* mice at postnatal day 28 (p28). Representative transverse sections are shown in Fig. 6A-C. WT muscles (Fig. 6A) exhibited minimal collagen deposition, consistent with healthy tissue architecture. Vehicle-treated *mdx* muscles (Fig. 6B) and PK007-treated *mdx* muscles (Fig. 6C) showed similar patterns of collagen deposition, with no marked increase in fibrosis.

Quantitative analysis of collagen deposition, expressed as a percentage of the total muscle cross-sectional area, further supports these observations (Fig. 6D-F). Across all analysed muscles, fibrosis levels were low and not significantly different between WT, Vehicle-treated *mdx*, and PK007-treated *mdx* mice (*p* > 0.05). These findings indicate that fibrosis is not a prominent feature of *mdx* muscles at p28, consistent with the acute phase of *mdx* mice, where muscle damage and inflammation are the predominant pathological features. This underscores the temporal progression of DMD, where fibrosis develops later during the chronic phase as a consequence of sustained cycles of damage and repair [34].

### Macrophages visualised with IBA-1 antibody are present at myonecrotic areas in mdx hindlimb muscles, with significantly fewer macrophages in PK007-treated mdx mice

**Fig. 7:**
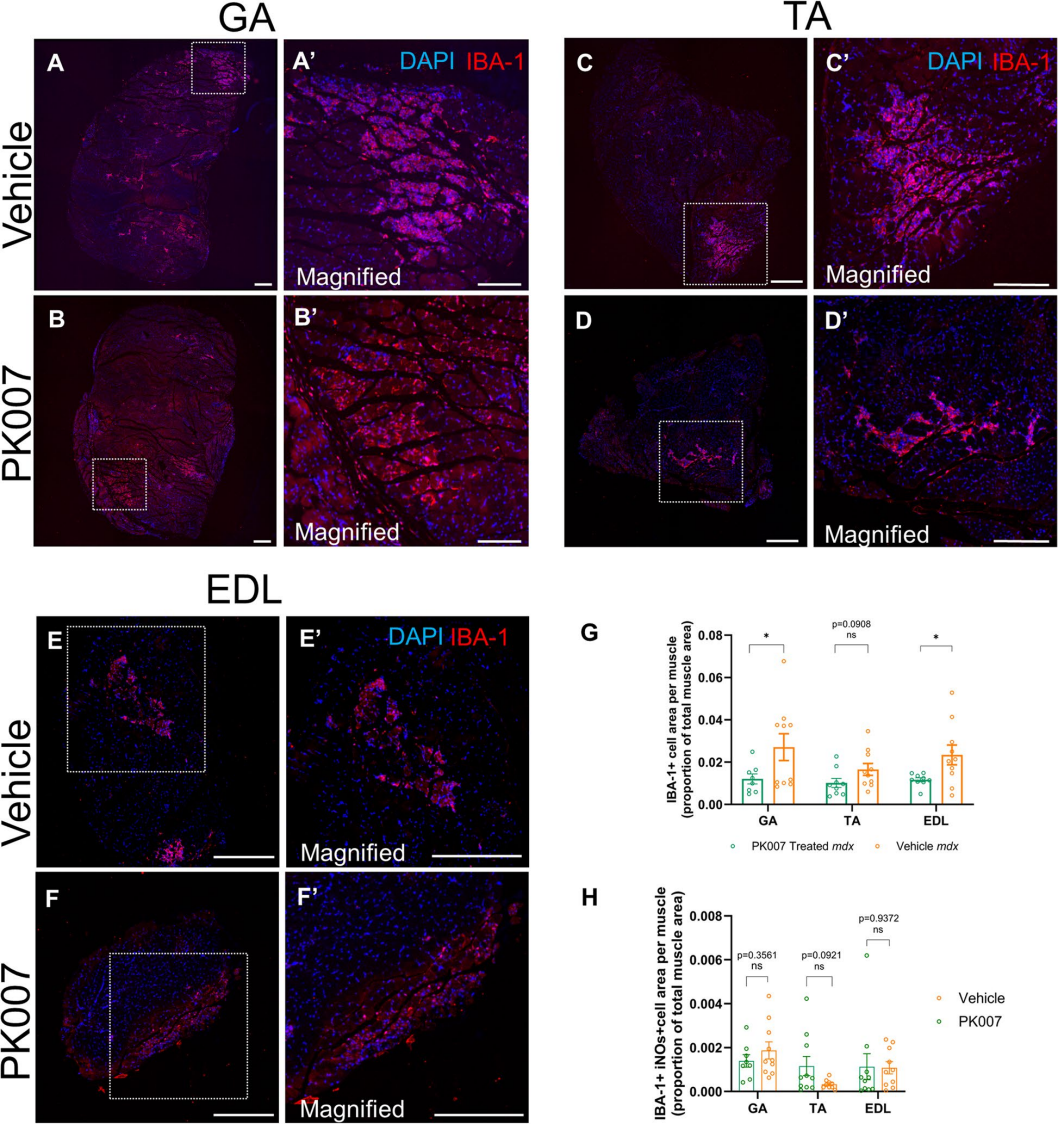
IBA-1 positive cell area in PK007 treated GA and EDL *mdx* muscles is significantly decreased. Representative images of IBA-1-labelled macrophages (red) with DAPI-labelled nuclei (blue) in PK007 and vehicle-treated *mdx* mice for the 3 hindlimb muscles; GA (**A**, **B**), TA (**C**, **D**) and TA (**E**, **F**). Scale bars 10 µm. Quantification (**G**) shows the average IBA-1-positive cell area per muscle (proportion of total muscle area) (mean ± SEM). Panel (**H**) represents the quantification of IBA-1 + /iNOS + macrophage area (M1 macrophage area) in the GA, TA, and EDL muscles of PK007- and vehicle-treated *mdx* mice. A Student’s *t*-test was performed between PK007 treated (green bars; GA: *n* = 10, TA: *n* = 10, EDL: *n* = 10) and vehicle-treated (orange bars; GA: *n* = 8, *p* = 0.0475; TA: *n* = 9, *p* = 0.0908; EDL: *n* = 9, *p* = 0.0342) to determine significance. Images were captured at 20 × objective on the Leica DMi8 SP8 inverted widefield microscope and magnified for quantification

It is known that macrophages play a pivotal role in exacerbating muscle cell lysis and resulting in myonecrosis in DMD [43, 61, 71, 78]. Quantification (by immunofluorescence) of the areas of macrophages (stained for IBA-1) shows images for GA, TA and EDL muscles for vehicle-treated and PK007-treated *mdx* mice (Figs. 7A to 7F). All muscles showed macrophages associated with sites of myonecrosis. Quantification of the IBA-1 positive cell area, proportional to the total muscle area for each hindlimb muscle (Fig. 7G), shows significantly less IBA-1 positive macrophage area in the PK007-treated GA and EDL muscles (0.012 ± 0.002 and 0.012 ± 0.001 respectively) compared to Vehicle-treated *mdx* mice (0.027 ± 0.006 and 0.023 ± 0.005 respectively). In TA muscles, there was a trend towards a reduced IBA-1positive cell area in PK007-treated *mdx* mice (0.017 ± 0.003) compared with Vehicle-treated *mdx* mice (0.010 ± 0.002), but this trend was not significant (*p* = 0.0908, *n* = 9). Further analysis of macrophage polarization (Fig. 7H) assessed the area occupied by IBA-1 + /iNOS + (M1) macrophages. Quantification showed no significant differences in M1 macrophage area between PK007- and Vehicle-treated *mdx* mice across all three muscles (GA: *p* = 0.3561; TA: *p* = 0.0921; EDL: *p* = 0.9372). These observations reveal that macrophage cell area was significantly decreased in the skeletal hindlimb muscles of PK007-treated *mdx* mice, but did not affect the polarization of macrophages towards the M1 phenotype. This reduction could be one mechanism contributing to the decreased myonecrotic area and damage that we have documented in this muscle (Fig. 5), which, in turn, could lead to increased muscle strength and movement being observed in PK007-treated *mdx* mice.

### PK007 reduces pro-inflammatory cytokine expression in GA and TA mdx muscles

In DMD, elevated levels of pro-inflammatory cytokines, such as iNOS, TNF-α, and IL-1β, contribute to acute inflammation and exacerbate muscle damage [18]. Targeting these cytokines offers a potential strategy to reduce inflammation-driven muscle necrosis and improve muscle health. To assess the anti-inflammatory effects of PK007, we evaluated the expression of iNOS, TNF-α, and IL-1β in the *gastrocnemius* (GA) and *tibialis anterior* (TA) muscles of *mdx* mice using immunofluorescence staining and quantitative analysis.

**Fig. 8:**
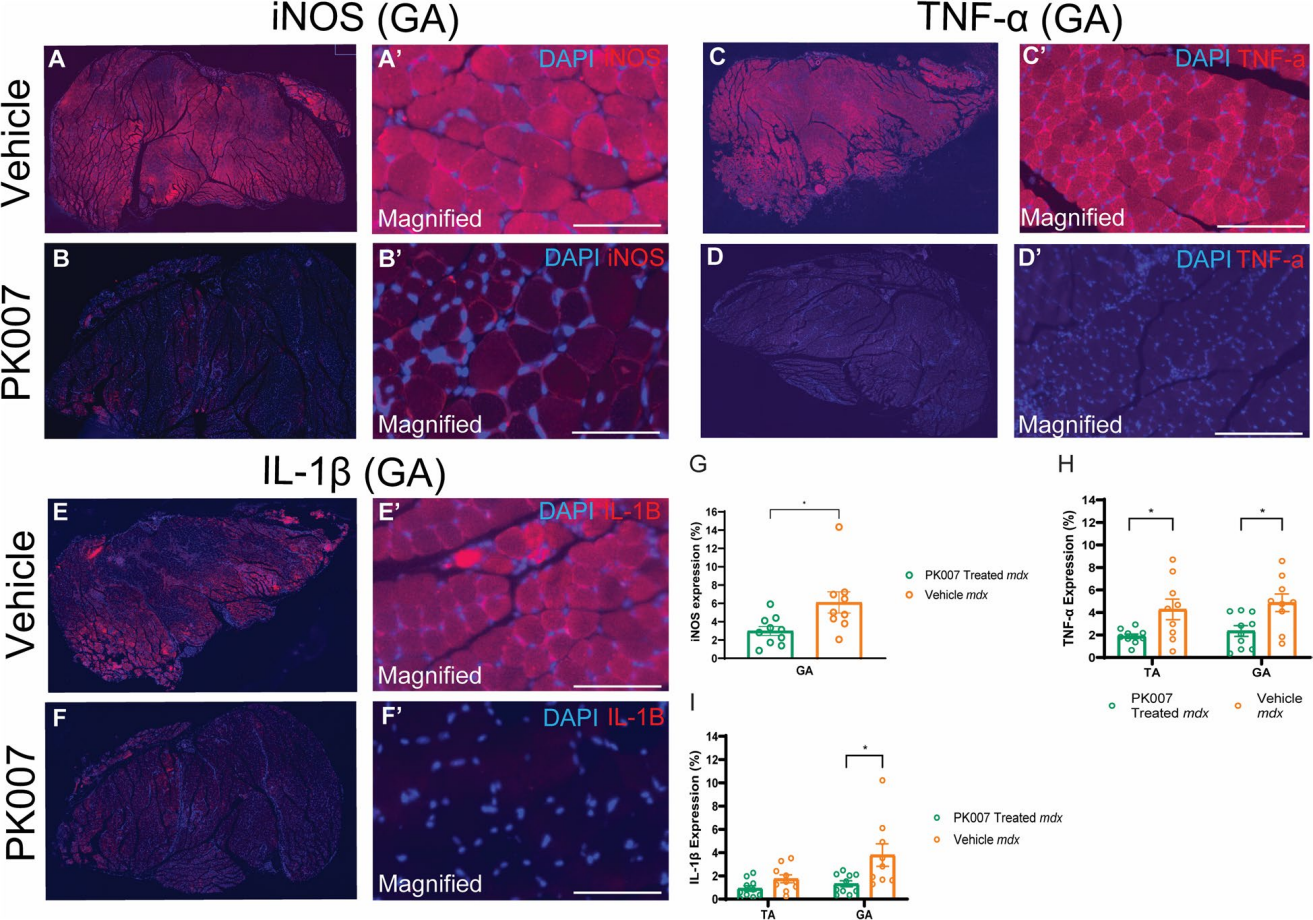
Pro-inflammatory markers, iNOS, TNF-α and IL-1β are decreased in PK007 treated *mdx* mice. Representative images of GA muscles from vehicle-treated (**A**, **C**, **E**) and PK007-treated (**B**, **D**, **F**) *mdx* mice, stained for pro-inflammatory markers iNOS, TNF-α, and IL-1β. Panels **A**-**B**’ show iNOS expression (red) with nuclei counterstained with DAPI (blue). Panels **C-D**’ show TNF-α expression (red) with DAPI counterstaining (blue), and panels **E**–**F**’ show IL-1β expression (red) with DAPI counterstaining (blue). Magnified regions (**A**’-**F**’) highlight reduced expression of pro-inflammatory markers in PK007-treated muscles. Quantitative analysis of marker expression is shown in panels **G-I**. Panel **G** depicts iNOS expression as a percentage of total muscle area, with significantly reduced levels in PK007-treated *mdx* muscles compared to Vehicle-treated controls (*p* = 0.0201). Panel** H** shows TNF-α expression percentages in the TA and GA muscles, demonstrating significant reductions in PK007-treated mice (*p* = 0.0156 for TA and *p* = 0.0181 for GA muscles). Panel **I** quantifies IL-1β expression percentages in TA and GA muscles, showing significant reductions in the GA muscle of PK007-treated mice compared to controls (*p* = 0.0593 for TA and *p* = 0.0197 for GA muscles). Data are presented as mean ± SEM, with individual points representing biological replicates, *n* = 10 in PK007-treated mdx mice and *n* = 9 in vehicle-treated *mdx* mice. Statistical significance was determined using unpaired two-tailed *t-*tests

Representative images of iNOS staining in the GA muscle (Fig. 8, A-B’) show reduced expression in PK007-treated muscles compared to Vehicle-treated controls. Quantitative analysis revealed that PK007 significantly decreased iNOS expression as a percentage of total muscle area in the GA muscle (*p* = *0.0201*; Fig. 8G). Specifically, the mean iNOS expression in PK007-treated *mdx* mice was 2.99 ± 0.49% (*n* = 10), compared to 6.11 ± 1.16% in vehicle-treated controls (*n* = 9). This significant reduction highlights the anti-inflammatory effect of PK007 on macrophage-related nitric oxide production. TNF-α staining in the TA and GA muscles (Fig. 8, C-D’) also showed reduced expression in PK007-treated mice. Quantitative analysis demonstrated significant decreases in TNF-α expression percentages in both the TA (*p* = *0.0156*) and GA (*p* = *0.0181*) muscles (Fig. 8H). The TA muscle’s mean TNF-α expression was 1.88 ± 0.20% in PK007-treated mice (*n* = 10) compared to 4.27 ± 0.91% in vehicle-treated mice (*n* = 9). Similarly, in the GA muscle, PK007-treated mice exhibited mean TNF-α expression of 2.35 ± 0.47%, compared to 4.86 ± 0.78% in vehicle-treated mice. These findings suggest that PK007 effectively reduces key pro-inflammatory cytokine production in DMD muscles. IL-1β staining was examined in the TA and GA muscles (Fig. 8, E–F’). Quantitative analysis showed significant reductions in IL-1β expression in the GA muscle of PK007-treated *mdx* mice (*p* = *0.0197*; Fig. 8I). Mean IL-1β expression in the GA muscle was 1.32 ± 0.26% in PK007-treated mice (*n* = 10), compared to 3.79 ± 0.97% in Vehicle-treated controls (*n* = 9). Although a trend of reduced IL-1β expression was observed in the TA muscle, this was not statistically significant (*p* = *0.0593*). Mean IL-1β expression in the TA muscle was 0.91 ± 0.24% for PK007-treated mice (*n* = 10) and 1.74 ± 0.34% for Vehicle-treated mice (*n* = 10).

Together, these results demonstrate that PK007 significantly reduces the expression of pro-inflammatory cytokines, including iNOS, TNF-α, and IL-1β, particularly in the GA and TA *mdx* muscles. This reduction in pro-inflammatory markers aligns with the observed decrease in muscle necrosis, suggesting that PK007 effectively attenuates inflammation during the acute phase of DMD.

## Discussion

Previous literature [32, 74] has supported the idea of reducing PGD2 to decrease muscle inflammation in *mdx* mice; PGD2 signalling promotes the recruitment of immune cells such as macrophages, mast cells and TH2 cells, thereby intensifying muscle damage and myonecrosis [31]. Further, previous HPGDS inhibitors such as HQL-79 and TAS-205 have reduced PGD2 and tetranor-PGDM levels while reducing necrotic muscle fibres and recovering the locomotor activity in *mdx* mice [54, 70]. Our previous preliminary research indicated that our HPGDS inhibitor, PK007, decreases myonecrosis and retains mature (healthy) muscle fibres in *mdx* mice [84]. The present study extends these early observations by exploring the pharmacokinetics of PK007 and further evaluating more diverse therapeutic benefits of PK007 by assessing muscle grip strength, total movement activity, myonecrosis and macrophage presence in 3 hindlimb muscle plus diaphragm and tongue muscles during a 10-day treatment period in juvenile *mdx* mice, sampled at 28 days postnatal. We have shown that by specifically decreasing PGD2 levels in *mdx* mice, muscle function (grip strength and locomotor activity) was significantly increased with PK007 treatment. This was likely due to the significant decrease in myonecrosis in hindlimb muscles (GA, TA, and EDL) and the diaphragm muscles we observed in the PK007-treated cohort. Additionally, we assessed the presence of macrophages in hindlimb muscles, which have been reported to exacerbate pathogenesis in the acute phase of DMD. We discovered a significant reduction in macrophage cell area in the GA and EDL muscles, which could explain the underlying decreased muscle inflammation.

### In vitro and in vivo assessments of potency and pharmacokinetics of PK007 highlighta significant decrease in PGD2 levels

The FP enzyme-based assay was the first tool in the potency assessment of our novel HPGDS inhibitor, PK007; this assay had a robust throughput [25]. Figure 1A outlined the FP IC50 curve of PK007, and a potency of 1.54 ± 0.26 µM was determined. Whilst this assay was suitable for a general indication of potency, it was necessary to further quantify the cell-based effect of PK007 before additional characterisation in vivo. To do this, we quantified the degree of PGD2 production in RAW 264.7 macrophage cell supernatant after treatment with PK007; similar assays have been previously conducted [56]. A dose-dependent inhibitory effect was observed on PGD2 generation (Fig. 1B). These data indicated the nanomolar potency of PK007 (17.23 ± 12 nM), 89-fold higher than when assessed via the FP assay. We confirmed that the dose-dependent reduction of PGD2 was due to the inhibitory effect of PK007 rather than macrophage cell death (Supplementary Fig. 3) as measured through an MTT assay [72]. Cells treated with PK007 displayed high survivability (> 100%) at 10 µM or lower concentrations. Similar nanomolar potency was seen from some HPGDS inhibitors synthesised and screened by Olsen and colleagues (2021) [56]; however, they have not reported further HPGDS inhibitor efficacy optimisation. Previously, literature reported that HPGDS inhibitor TAS-205 has been investigated in Guinea pig models of allergic rhinitis. Aoyagi et al. [4] determined the in vitro potency of TAS-205; the IC50 values were 181.3 nM and 78.3 nM in the rat (RBL-2H3) and human (KU812) basophilic cell lines, respectively [4]. Further studies should explore the potency of TAS-205 in RAW 264.7 macrophage cells to accurately compare the molecule to PK007 and elucidate the best-in-class HPGDS inhibitor.

To ensure PK007 has adequate exposure for in vivo efficacy studies, we assessed the pharmacokinetic properties in C57BL/6 male mice (Fig. 1C and Supplementary Table 1). These properties included low clearance (50.4 ± 4.8 mL/min/kg), high volume of distribution (7.3 ± 4.6 L/kg), and a moderate half-life (1.9 ± 0.3 h for IV; 3.0 ± 0.3 h for oral). Oral administration showed rapid absorption (C_max_ = 360 ± 16.4 nM within 0.25 h) and excellent bioavailability (81%). Taken with the cell-based potency data of Fig. 1B, we can further predict the required dosing regimen to inhibit PGD2 production over 24 h. Knowing that the IC50 of PK007 was 17.23 ± 12 nM (Fig. 1B), we can extrapolate the PO pharmacokinetic half-life to determine that PGD2 inhibition will occur up to approximately 13 h (Fig. 1C). Therefore, it is suggested that future studies explore the twice-daily oral dosing of the inhibitor rather than the once-administered dose, as conducted in Figs. 2, 3, 4, 5, 6, 7 and 8. Overall, the pharmacokinetic assessment of PK007 underpins its potential as a practical oral therapeutic, leading to further in vivo studies to optimise and validate its beneficial effect within *mdx* mice. For example, PK analyses in C57Bl/10 *mdx* mice will help confirm translatability under muscular dystrophy conditions.

Next, we evaluated PK007’s effectiveness in inhibiting the systemic production of PGD2 in the *mdx* model of DMD (p28). The PGD2-MOX ELISA displayed an approximately 33.36% reduction of PGD2 concentration levels in the PK007 cohort, a significant decrease compared to the Vehicle-treated cohort. This further confirms, alongside our in vitro data, that PK007 successfully inhibits HPGDS and decreases PGD2 levels.

### PK007-treated mdx mice display improved muscle function comparable to Prednisone-treated and wild-type mice and enhanced locomotor activity

Previous studies have demonstrated various degrees of success in enhancing muscle strength and function, which are primary symptoms of DMD. For instance, a study by Mohri et al. [54] showed that inhibition of hematopoietic prostaglandin D synthase (HPGDS) with HQL-79 resulted in an approximately 23% increase in hindlimb muscle strength in *mdx* mice [54]. By contrast, our research has shown a more significant improvement, with a 50% increase in grip strength in *mdx* mice. This enhancement is potentially due to the increased potency of PK007 (17.23 ± 12 nM), considerably greater than the literature-reported compound, HQL-79 (6 μM) [6].

In addition to the observed improvements in muscle strength, our locomotor activity assessment further supports our treatment’s efficacy. We noted a significant increase in locomotor activity in PK007-treated *mdx* mice, indicating enhanced muscle function. This finding aligns with results from the clinical trial of the HPGDS inhibitor TAS-205, which demonstrated reduced muscle necrosis in hindlimb muscles and improved muscle function and movement in DMD patients [39, 69]. By contrast, current glucocorticoid treatments, while effective in improving muscle strength, have not shown improvements in locomotor activity [57]. Moreover, prolonged use of glucocorticoids is associated with adverse effects such as reduced bone density, weight gain, and growth suppression, all of which negatively impact ambulation and overall movement [50, 58, 60, 68, 85]. This comparison highlights the potential advantages of PK007 treatment for reduced myonecrosis, enhancing muscle strength, and improving overall motor function.

It has been well documented that DMD patients and *mdx* mice typically exhibit significantly reduced aerobic capacity in comparison to healthy individuals and WT mice, respectively [36, 48, 64, 65]. This observation is primarily due to the progressive loss of skeletal muscle strength and function, a primary hallmark of the disease [49, 65]. As respiratory muscles such as the diaphragm weaken in DMD, they impair effective breathing, reducing oxygen intake and exacerbating muscle weakness [11, 16, 33, 64]. Our findings confirmed that *mdx* mice exhibit lower VO_2_ levels than WT mice (Fig. 4; approximately 35.84% lower), consistent with published literature [29, 62]. Interestingly, PK007 treatment did not significantly alter aerobic capacity in *mdx* mice over the 10-day treatment period compared to vehicle treatment. It is important to note that these assessments did not include an exercise regimen, as exercise provides a more precise measurement of aerobic capacity under stress conditions. Future studies should evaluate the effects of PK007 over longer durations and at different time points of the disease to determine whether it impacts aerobic capacity and muscle strength during progressive stages of DMD. Incorporating exercise protocols in these studies would further elucidate the therapeutic potential of PK007 under conditions that mimic physiological stress [29].

### The total myonecrotic area in diverse PK007-treated muscles significantly decreased in mdx mice

Prior research has highlighted PGD2 as a pivotal enzyme exacerbating muscle damage and contributing to myonecrosis [30, 56]. Our study revealed that inhibiting PGD2 with PK007 reduced myonecrosis, potentially explaining the muscle strength and mobility improvements observed in PK007-treated *mdx* mice. These findings are consistent with the findings of other researchers and our initial studies, where HPGDS inhibition has led to improved muscle function in *mdx* mice during the chronic disease stage [54] and earlier during the acute disease phase, where muscle damage is overt [84].

To further confirm the reduction in muscle damage, we employed IgG staining as a marker of muscle fibre membrane permeability (Supplementary Fig. 4), which indicates necrotic or damaged fibres in DMD (Dubuisson et al., 2022). Elevated IgG infiltration correlates with areas of active myonecrosis, indicating muscle injury. In PK007-treated *mdx* muscles, IgG staining revealed a significant reduction in damaged regions compared to Vehicle-treated controls. This reduction was most evident in the TA muscle, where IgG-positive areas in PK007-treated *mdx* mice approached levels observed in WT-vehicle-treated mice. This finding further supports the role of PK007 in reducing muscle damage.

Our results reveal a novel reduction in myonecrosis, supported by histological analysis and IgG staining, specifically in the GA and EDL muscles. This outcome, not previously reported in studies of earlier HPGDS inhibitors, underscores the therapeutic potential of PK007 [39, 54]. By reducing muscle necrosis, PK007 likely contributes to the improvements in muscle strength and function reported in our study and others using HPGDS inhibitors. Future pre-clinical studies will aim to compare PK007 with TAS-205, currently in phase 3 clinical trials (NCT04587908), to evaluate their relative effectiveness and therapeutic outcomes in mitigating DMD progression.

The diaphragm and tongue muscles are known to be compromised in clinical DMD, with impairments in respiratory function and speech becoming more pronounced in the later stages of the disease [16, 33] [76, 82]. It has been reported that degeneration of the phrenic and hypoglossal (XII) nerves plays a significant role in the dysfunction of these muscles. A study by Dhindsa et al. [19] revealed that the phrenic nerve, as the sole motor innervation to the diaphragm, is vital for breathing, and its degeneration leads to respiratory failure, the most common cause of death in DMD patients [12, 19]. The diaphragm, constantly engaged due to its role in breathing, suffers from chronic mechanical stress, resulting in marked muscle loss, fibrosis, and eventual respiratory insufficiency. Similarly, the hypoglossal nerve, responsible for motor control of the tongue, is crucial for speech and swallowing [19]. Although the tongue has been underexplored in *mdx* models [12, 19], clinical reports show that dysphagia, or difficulty swallowing, is prevalent among DMD boys, likely linked to this neuromuscular degeneration [19]. These findings reveal the importance of studying the diaphragm and tongue muscles to understand the progression of DMD and potential therapeutic interventions. This study aimed to examine if these muscles during the acute phase were also affected similarly to that seen for leg muscles and, if so, the impact of early HPGDS inhibitor intervention. We observed areas of myonecrosis in both the tongue and diaphragm muscles in vehicle-treated *mdx* mice similar to that reported in the chronic disease phase in *mdx* DMD model mice [20, 46]. Our inhibition of HPDGS by PK007 significantly reduced myonecrotic areas in both the tongue and the diaphragm during an early treatment period from postnatal day (p) 18 to p28 – a period that models the acute phase of DMD [2], suggesting that early HPGDS inhibitor intervention could also assist in reducing dysfunction of these specific muscles in DMD.

### The reduction of myonecrosis and pro-inflammatory markers correlates with a decreased macrophage cell population in PK007-treated mdx mice

In DMD, macrophages aggravate the disease’s progression [5, 81]. For example, DMD muscle-resident macrophages express high levels of matrix metalloprotease-9 (MMP-9), which leads to the overproduction of pro-inflammatory cytokines such as IFN-γ, NF-κB, IL-1β and IL-6 [21]. These cytokines, in turn, increase oxidative stress and promote the secretion of TGF-β [24]. Additionally, M1 macrophages are known to enhance the secretion of TNF-α and activate necrotic pathways dependent on receptor-interacting protein kinase-1 [22]. They also inhibit IL-4 and block the proliferation of M2 macrophages, which are crucial for skeletal muscle regeneration [75]. These combined effects significantly contribute to muscle degeneration and weakness in DMD [43, 67, 74, 77, 80, 81].

PGD2 is implicated in exacerbating the pathogenesis of DMD by recruiting macrophages to sites of muscle damage, where these immune cells further contribute to inflammation and tissue degeneration [5, 43, 80]. PGD2 promotes the migration and activation of macrophages by binding to its receptors, DP1 and DP2, on the surface of these immune cells [54]. This binding initiates a signalling cascade that enhances the inflammatory response and perpetuates muscle damage [54].

In this study, we demonstrated that PK007, a novel inhibitor of PGD2, significantly reduced macrophage area within the hind limb muscles of treated *mdx* mice compared to Vehicle-treated controls. In addition to reducing macrophage area, PK007 treatment decreased the expression of key pro-inflammatory cytokines, including iNOS, TNF-α, and IL-1β, in the gastrocnemius muscle (Fig. 8). By contrast, macrophage polarization toward the M1 phenotype remained unaffected**.** These findings suggest that PK007 primarily decreases pro-inflammatory signalling and inflammation without altering the polarization state of macrophages. This reduction in pro-inflammatory cytokine expression, combined with a decreased macrophage area population, likely prevents further muscle injury and progression to myonecrosis. By decreasing PGD2 levels, PK007 inhibits the signalling pathways that recruit macrophages while reducing the inflammatory cytokine burden. Similar effects have been observed with the HPGDS inhibitor HQL-79, suggesting that PK007 can prevent further muscle injury by reducing the presence of inflammatory immune cells [54]. In future, we aim to extend our macrophage and cytokine investigation to the tongue and diaphragm dystrophic muscles, where we observed a decrease in necrosis, to assess whether the pathogenesis is consistent with that of skeletal limb muscles.

In conclusion, currently, no viable long-term therapy exists for DMD, yet there is compelling evidence that persistent inflammation exacerbates myonecrosis and disease progression [32]. Studies have identified a critical role for prostaglandins, particularly PGD2, in mediating inflammatory responses that contribute to DMD progression [32]. Here, we have described our novel HPGDS inhibitor, PK007, demonstrating nanomolar potency and excellent oral pharmacokinetics. Our study has shown that in orally administered PK007 in *mdx* mice, decreased PGD2 levels correlated with reduced myonecrotic area and reduced macrophages, with improved muscle function and movement. Thus, it seems likely that the one major outcome of the PK007-decreased PGD2 concentration is a reduction in macrophage numbers, which are known to exacerbate myonecrosis in DMD [74]. These findings underscore the critical role of PGD2 in the pathophysiology of DMD and highlight the efficacy of targeted intervention strategies that disrupt this pathway (e.g., using PK007), offering a promising avenue for future treatments to reduce disease severity (and help maintain muscle mass) in young DMD boys.

## Supplementary Information

Supplementary Material 1.

## Data Availability

No datasets were generated or analysed during the current study.

## References

[1] Aartsma-Rus A, van Putten M. Assessing functional performance in the *mdx* mouse model. J Vis Exp. 2014;(85). 10.3791/51303 10.3791/51303 PMC4158772 24747372

[2] Al-Mshhdani BA, Grounds MD, Arthur PG, Terrill JR. A Blood Biomarker for Duchenne Muscular Dystrophy Shows That Oxidation State of Albumin Correlates with Protein Oxidation and Damage in *Mdx* Muscle. Antioxidants (Basel). 2021;10(8). 10.3390/antiox10081241 10.3390/antiox10081241 PMC8389308 34439489

[3] Ananthasubramaniam B, Meijer JH. Regulation of Rest, Rather Than Activity, Underlies Day-Night Activity Differences in Mice. Front Physiol. 2020;11:268. 10.3389/fphys.2020.00268. 32296342 10.3389/fphys.2020.00268 PMC7136415

[4] Aoyagi H, Kajiwara D, Tsunekuni K, Tanaka K, Miyoshi K, Hirasawa N. Potential synergistic effects of novel hematopoietic prostaglandin D synthase inhibitor TAS-205 and different types of anti-allergic medicine on nasal obstruction in a Guinea pig model of experimental allergic rhinitis. Eur J Pharmacol. 2020;875:173030. 10.1016/j.ejphar.2020.173030. 32084417 10.1016/j.ejphar.2020.173030

[5] Arima M, Fukuda T. Prostaglandin D(2) and T(H)2 inflammation in the pathogenesis of bronchial asthma. Korean J Intern Med. 2011;26(1):8–18. 10.3904/kjim.2011.26.1.8. 21437156 10.3904/kjim.2011.26.1.8 PMC3056260

[6] Aritake K, Kado Y, Inoue T, Miyano M, Urade Y. Structural and functional characterization of HQL-79, an orally selective inhibitor of human hematopoietic prostaglandin D synthase. J Biol Chem. 2006;281(22):15277–86. 10.1074/jbc.M506431200. 16547010 10.1074/jbc.M506431200

[7] Bains RS, Wells S, Sillito RR, Armstrong JD, Cater HL, Banks G, Nolan PM. Assessing mouse behaviour throughout the light/dark cycle using automated in-cage analysis tools. J Neurosci Methods. 2018;300:37–47. 10.1016/j.jneumeth.2017.04.014. 28456660 10.1016/j.jneumeth.2017.04.014 PMC5909039

[8] Bankhead P, Loughrey MB, Fernandez JA, Dombrowski Y, McArt DG, Dunne PD, McQuaid S, Gray RT, Murray LJ, Coleman HG, James JA, Salto-Tellez M, Hamilton PW. QuPath: Open source software for digital pathology image analysis. Sci Rep. 2017;7(1):16878. 10.1038/s41598-017-17204-5. 29203879 10.1038/s41598-017-17204-5 PMC5715110

[9] Barr JT, Tran TB, Rock BM, Wahlstrom JL, Dahal UP. Strain-Dependent Variability of Early Discovery Small Molecule Pharmacokinetics in Mice: Does Strain Matter? Drug Metab Dispos. 2020;48(8):613–21. 10.1124/dmd.120.090621. 32474442 10.1124/dmd.120.090621

[10] Angulski BB, A., Hosny, N., Cohen, H., Martin, A. A., Hahn, D., Bauer, J., & Metzger, J. M. Duchenne muscular dystrophy: disease mechanism and therapeutic strategies. Front Physiol. 2023;14:1183101. 10.3389/fphys.2023.1183101. 37435300 10.3389/fphys.2023.1183101 PMC10330733

[11] Birnkrant DJ, Bushby K, Bann CM, Apkon SD, Blackwell A, Brumbaugh D, Case LE, Clemens PR, Hadjiyannakis S, Pandya S, Street N, Tomezsko J, Wagner KR, Ward LM, Weber DR. Diagnosis and management of Duchenne muscular dystrophy, part 1: diagnosis, and neuromuscular, rehabilitation, endocrine, and gastrointestinal and nutritional management. Lancet Neurol. 2018;17(3), 251–267. 10.1016/S1474-4422(18)30024-3 10.1016/S1474-4422(18)30024-3 PMC5869704 29395989

[12] Chamberlain JS, Metzger J, Reyes M, Townsend D, Faulkner JA. Dystrophin-deficient *mdx* mice display a reduced life span and are susceptible to spontaneous rhabdomyosarcoma. FASEB J. 2007;21(9):2195–204. 10.1096/fj.06-7353.com. 17360850 10.1096/fj.06-7353com

[13] Chang M, Cai Y, Gao Z, Chen X, Liu B, Zhang C, Yu W, Cao Q, Shen Y, Yao X, Chen X, Sun H. Duchenne muscular dystrophy: pathogenesis and promising therapies. J Neurol. 2023;270(8):3733–49. 10.1007/s00415-023-11796-x. 37258941 10.1007/s00415-023-11796-x

[14] Cheung SW, Bhavnani E, Simmons DG, Bellingham MC, Noakes PG. Perineuronal nets are phagocytosed by MMP-9 expressing microglia and astrocytes in the SOD1(G93A) ALS mouse model. Neuropathol Appl Neurobiol. 2024;50(3):e12982. 10.1111/nan.12982. 38742276 10.1111/nan.12982

[15] Christ AN, Labzin L, Bourne GT, Fukunishi H, Weber JE, Sweet MJ, Smythe ML, Flanagan JU. Development and characterization of new inhibitors of the human and mouse hematopoietic prostaglandin D(2) synthases. J Med Chem. 2010;53(15):5536–48. 10.1021/jm100194a. 20684598 10.1021/jm100194a

[16] Clark AL. Origin of symptoms in chronic heart failure. Heart. 2006;92(1):12–6. 10.1136/hrt.2005.066886. 16159969 10.1136/hrt.2005.066886 PMC1860978

[17] Collins CA, Morgan JE. Duchenne’s muscular dystrophy: animal models used to investigate pathogenesis and develop therapeutic strategies. Int J Exp Pathol. 2003;84(4):165–72. 10.1046/j.1365-2613.2003.00354.x. 14632630 10.1046/j.1365-2613.2003.00354.x PMC2517561

[18] De Paepe B, De Bleecker JL. Cytokines and chemokines as regulators of skeletal muscle inflammation: presenting the case of Duchenne muscular dystrophy. Mediators Inflamm. 2013;2013:540370. 10.1155/2013/540370. 24302815 10.1155/2013/540370 PMC3835490

[19] Dhindsa JS, McCall AL, Strickland LM, Fusco AF, Kahn AF, ElMallah MK. Motor axonopathies in a mouse model of Duchenne muscular dystrophy. Sci Rep. 2020;10(1):8967. 10.1038/s41598-020-65824-1. 32488044 10.1038/s41598-020-65824-1 PMC7265344

[20] da Silva HNM, Covatti C, da Rocha GL, Mizobuti DS, Mancio RD, Hermes TA, Kido LA, Cagnon VHA, Pereira ECL, Minatel E. Oxidative Stress, Inflammation, and Activators of Mitochondrial Biogenesis: Tempol Targets in the Diaphragm Muscle of Exercise Trained-*mdx* Mice. Front Physiol. 2021;12:649793. 10.3389/fphys.2021.649793. 33981250 10.3389/fphys.2021.649793 PMC8107395

[21] Dixit VD, Yang H, Cooper-Jenkins A, Giri BB, Patel K, Taub DD. Reduction of T cell-derived ghrelin enhances proinflammatory cytokine expression: implications for age-associated increases in inflammation. Blood. 2009;113(21):5202–5. 10.1182/blood-2008-09-181255. 19324904 10.1182/blood-2008-09-181255 PMC2686189

[22] Duan D. Systemic AAV Micro-dystrophin Gene Therapy for Duchenne Muscular Dystrophy. Mol Ther. 2018;26(10):2337–56. 10.1016/j.ymthe.2018.07.011. 30093306 10.1016/j.ymthe.2018.07.011 PMC6171037

[23] Duan D, Goemans N, Takeda S, Mercuri E, Aartsma-Rus A. Duchenne muscular dystrophy. Nat Rev Dis Primers. 2021;7(1):13. 10.1038/s41572-021-00248-3. 33602943 10.1038/s41572-021-00248-3 PMC10557455

[24] Dunkelberger JR, Song WC. Complement and its role in innate and adaptive immune responses. Cell Res. 2010;20(1):34–50. 10.1038/cr.2009.139. 20010915 10.1038/cr.2009.139

[25] Edfeldt F, Evenäs J, Lepistö M, Ward A, Petersen J, Wissler L, Rohman M, Sivars U, Svensson K, Perry M, Feierberg I, Zhou XH, Hansson T, Narjes F. Identification of indole inhibitors of human hematopoietic prostaglandin D2 synthase (hH-PGDS). Bioorganic Med Chemi Letters. 2015;25(12):2496–2500. 10.1016/j.bmcl.2015.04.065 10.1016/j.bmcl.2015.04.065 25978964

[26] Grounds M. (2010, 25/01/2014). Quantification of histopathology in Haemotoxylin and Eosin stained muscle sections. https://www.treat-nmd.org/wp-content/uploads/2023/07/MDX-DMD_M.1.2.007-28.pdf

[27] Grounds MD, Lloyd EM. Considering the Promise of Vamorolone for Treating Duchenne Muscular Dystrophy. J Neuromuscul Dis. 2023;10(6):1013–30. 10.3233/JND-230161. 37927274 10.3233/JND-230161 PMC10657680

[28] Grounds MD, Terrill JR, Al-Mshhdani BA, Duong MN, Radley-Crabb HG, Arthur PG. Biomarkers for Duchenne muscular dystrophy: myonecrosis, inflammation and oxidative stress. Dis Model Mech. 2020;13(2). 10.1242/dmm.043638 10.1242/dmm.043638 PMC7063669 32224496

[29] Hafstad AD, Boardman NT, Lund J, Hagve M, Khalid AM, Wisloff U, Larsen TS, Aasum E. High intensity interval training alters substrate utilization and reduces oxygen consumption in the heart. J Appl Physiol (1985). 2011;111(5):1235–1241. 10.1152/japplphysiol.00594.2011 10.1152/japplphysiol.00594.2011 21836050

[30] Hamamura K, Yoshida Y, Oyama K, Li J, Kawano S, Inoue K, Toyooka K, Yamadera M, Matsunaga N, Matsumura T, Aritake K. Hematopoietic Prostaglandin D Synthase Is Increased in Mast Cells and Pericytes in Autopsy Myocardial Specimens from Patients with Duchenne Muscular Dystrophy. Int J Mol Sci. 2021;25(3). 10.3390/ijms25031846 PMC10855661 38339125

[31] Herbelet S, Rodenbach A, Paepe B, De Bleecker JL. Anti-Inflammatory and General Glucocorticoid Physiology in Skeletal Muscles Affected by Duchenne Muscular Dystrophy: Exploration of Steroid-Sparing Agents. Int J Mol Sci. 2020;21(13). 10.3390/ijms21134596 10.3390/ijms21134596 PMC7369834 32605223

[32] Hoxha M. Duchenne muscular dystrophy: Focus on arachidonic acid metabolites. Biomed Pharmacother. 2019;110:796–802. 10.1016/j.biopha.2018.12.034. 30554118 10.1016/j.biopha.2018.12.034

[33] Katz SD, Zheng H. Peripheral limitations of maximal aerobic capacity in patients with chronic heart failure. J Nucl Cardiol. 2002;9(2):215–25. 10.1067/mnc.2002.123183. 11986567 10.1067/mnc.2002.123183

[34] Kharraz Y, Guerra J, Pessina P, Serrano AL, Munoz-Canoves P. Understanding the process of fibrosis in Duchenne muscular dystrophy. Biomed Res Int. 2014;2014:965631. 10.1155/2014/965631. 24877152 10.1155/2014/965631 PMC4024417

[35] Kilkenny C, Browne W, Cuthill IC, Emerson M, Altman DG, Group NCRRGW. Animal research: reporting in vivo experiments: the ARRIVE guidelines. Br J Pharmacol. 2010;160(7):1577-1579. 10.1111/j.1476-5381.2010.00872.x 10.1111/j.1476-5381.2010.00872.x PMC2936830 20649561

[36] Kim JH, Kwak HB, Thompson LV, Lawler JM. Contribution of oxidative stress to pathology in diaphragm and limb muscles with Duchenne muscular dystrophy. J Muscle Res Cell Motil. 2013;34(1):1–13. 10.1007/s10974-012-9330-9. 23104273 10.1007/s10974-012-9330-9

[37] Klingler W, Jurkat-Rott K, Lehmann-Horn F, Schleip R. The role of fibrosis in Duchenne muscular dystrophy. Acta Myol, 2012;31(3):184–195. https://www.ncbi.nlm.nih.gov/pubmed/23620650 PMC3631802 23620650

[38] Kohler M, Clarenbach CF, Bahler C, Brack T, Russi EW, Bloch KE. Disability and survival in Duchenne muscular dystrophy. J Neurol Neurosurg Psychiatry. 2009;80(3):320–5. 10.1136/jnnp.2007.141721. 18713792 10.1136/jnnp.2007.141721

[39] Komaki H, Maegaki Y, Matsumura T, Shiraishi K, Awano H, Nakamura A, Kinoshita S, Ogata K, Ishigaki K, Saitoh S, Funato M, Kuru S, Nakayama T, Iwata Y, Yajima H, Takeda S. Early phase 2 trial of TAS-205 in patients with Duchenne muscular dystrophy. Ann Clin Transl Neurol. 2020;7(2):181–90. 10.1002/acn3.50978. 31957953 10.1002/acn3.50978 PMC7034509

[40] Kourakis S, Timpani CA, Campelj DG, Hafner P, Gueven N, Fischer D, Rybalka E. Standard of care versus new-wave corticosteroids in the treatment of Duchenne muscular dystrophy: Can we do better? Orphanet J Rare Dis. 2021;16(1):117. 10.1186/s13023-021-01758-9. 33663533 10.1186/s13023-021-01758-9 PMC7934375

[41] Lea TA, Panizza PM, Arthur PG, Bakker AJ, Pinniger GJ. Hypochlorous acid exposure impairs skeletal muscle function and Ca(2+) signalling: implications for Duchenne muscular dystrophy pathology. J Physiol. 2023;601(23):5257–75. 10.1113/JP285263. 37864413 10.1113/JP285263

[42] Lemos JP, Tenorio LPG, Mouly V, Butler-Browne G, Mendes-da-Cruz DA, Savino W, Smeriglio P. T cell biology in neuromuscular disorders: a focus on Duchenne Muscular Dystrophy and Amyotrophic Lateral Sclerosis. Front Immunol. 2023;14:1202834. 10.3389/fimmu.2023.1202834. 37920473 10.3389/fimmu.2023.1202834 PMC10619758

[43] Lendeckel U, Venz S, Wolke C. Macrophages: shapes and functions. ChemTexts. 2022;8(2):12. 10.1007/s40828-022-00163-4. 35287314 10.1007/s40828-022-00163-4 PMC8907910

[44] Lindell DM, Lukacs NW. Cytokines and Chemokines in Inflammation. In C. N. Serhan, P. A. Ward, & D. W. Gilroy (Eds.), Fundamentals of Inflammation (pp. 175–185). Cambridge University Press. 2010. 10.1017/CBO9781139195737.015

[45] Liu T, Zhang L, Joo D, Sun SC. NF-kappaB signaling in inflammation. Signal Transduct Target Ther. 2017;2:17023-. 10.1038/sigtrans.2017.23 10.1038/sigtrans.2017.23 PMC5661633 29158945

[46] Lorena M, Santos EKD, Ferretti R, Nagana Gowda GA, Odom GL, Chamberlain JS, Matsumura CY. Biomarkers for Duchenne muscular dystrophy progression: impact of age in the *mdx* tongue spared muscle. Skelet Muscle. 2023;13(1):16. 10.1186/s13395-023-00325-z. 37705069 10.1186/s13395-023-00325-z PMC10500803

[47] Luca D. (2008, 2019). Use of grip strength meter to assess the limb strength of mdx mice. Retrieved 3/05 from https://www.treat-nmd.org/wp-content/uploads/2023/07/MDX-DMD_M.2.2.001.pdf

[48] Mayer OH. Clinical pulmonary function testing in Duchenne muscular dystrophy. Paediatr Respir Rev. 2019;30:9–12. 10.1016/j.prrv.2018.08.004. 30413352 10.1016/j.prrv.2018.08.004

[49] Mayer OH, Finkel RS, Rummey C, Benton MJ, Glanzman AM, Flickinger J, Lindstrom BM, Meier T. Characterization of pulmonary function in Duchenne Muscular Dystrophy. Pediatr Pulmonol. 2015;50(5):487–94. 10.1002/ppul.23172. 25755201 10.1002/ppul.23172 PMC4402127

[50] McDonald CM, Henricson EK, Abresch RT, Duong T, Joyce NC, Hu F, Clemens PR, Hoffman EP, Cnaan A, Gordish-Dressman H, Investigators C. Long-term effects of glucocorticoids on function, quality of life, and survival in patients with Duchenne muscular dystrophy: a prospective cohort study. Lancet. 2018;391(10119):451–61. 10.1016/S0140-6736(17)32160-8. 29174484 10.1016/S0140-6736(17)32160-8

[51] McGrath JC, Lilley E. Implementing guidelines on reporting research using animals (ARRIVE etc.): new requirements for publication in BJP. Br J Pharmacol. 2015;172(13):3189–3193. 10.1111/bph.12955 10.1111/bph.12955 PMC4500358 25964986

[52] Mercuri E, Bonnemann CG, Muntoni F. Muscular dystrophies. Lancet. 2019;394(10213):2025–38. 10.1016/S0140-6736(19)32910-1. 31789220 10.1016/S0140-6736(19)32910-1

[53] Milad N, White Z, Tehrani AY, Sellers S, Rossi FMV, Bernatchez P. Increased plasma lipid levels exacerbate muscle pathology in the *mdx* mouse model of Duchenne muscular dystrophy. Skeletal Muscle. 2017;7(1):19. 10.1186/s13395-017-0135-9. 28899419 10.1186/s13395-017-0135-9 PMC5596936

[54] Mohri I, Aritake K, Taniguchi H, Sato Y, Kamauchi S, Nagata N, Maruyama T, Taniike M, Urade Y. Inhibition of prostaglandin D synthase suppresses muscular necrosis. Am J Pathol. 2009;174(5):1735–44. 10.2353/ajpath.2009.080709. 19359520 10.2353/ajpath.2009.080709 PMC2671262

[55] Motulsky HJ, Brown RE. Detecting outliers when fitting data with nonlinear regression - a new method based on robust nonlinear regression and the false discovery rate. BMC Bioinformatics. 2006;7:123. 10.1186/1471-2105-7-123. 16526949 10.1186/1471-2105-7-123 PMC1472692

[56] Olson KL, Holt MC, Ciske FL, Kramer JB, Heiple PE, Collins ML, Johnson CM, Ho CS, Morano MI, Barrett SD. Novel amide and imidazole compounds as potent hematopoietic prostaglandin D2 synthase inhibitors. Bioorganic Med Chemi Letters. 2021;34:127759. 10.1016/j.bmcl.2020.127759 10.1016/j.bmcl.2020.127759 33383152

[57] Quattrocelli M, Salamone IM, Page PG, Warner JL, Demonbreun AR, McNally EM. Intermittent Glucocorticoid Dosing Improves Muscle Repair and Function in Mice with Limb-Girdle Muscular Dystrophy. Am J Pathol. 2017;187(11):2520–35. 10.1016/j.ajpath.2017.07.017. 28823869 10.1016/j.ajpath.2017.07.017 PMC5809598

[58] Quattrocelli M, Zelikovich AS, Salamone IM, Fischer JA, McNally EM. Mechanisms and Clinical Applications of Glucocorticoid Steroids in Muscular Dystrophy. J Neuromuscul Dis. 2021;8(1):39–52. 10.3233/JND-200556. 33104035 10.3233/JND-200556 PMC7902991

[59] Radley-Crabb HG, Marini JC, Sosa HA, Castillo LI, Grounds MD, Fiorotto ML. Dystropathology increases energy expenditure and protein turnover in the *mdx* mouse model of duchenne muscular dystrophy. PLoS ONE. 2014;9(2):e89277. 10.1371/journal.pone.0089277. 24586653 10.1371/journal.pone.0089277 PMC3929705

[60] Ramamoorthy S, Cidlowski,JA. Corticosteroids: Mechanisms of Action in Health and Disease. Rheum Dis Clin North Am. 2016;42(1):15–31, vii. 10.1016/j.rdc.2015.08.002 10.1016/j.rdc.2015.08.002 PMC4662771 26611548

[61] Rittchen S, Jandl K., Lanz, I., Reiter, B., Ferreirós, N., Kratz, D., Lindenmann, J., Brcic, L., Bärnthaler, T., Atallah, R., Olschewski, H., Sturm, E. M., & Heinemann, A. Monocytes and Macrophages Serve as Potent Prostaglandin D 2 Sources during Acute, Non-Allergic Pulmonary Inflammation. Int J Mol Sci.. 2021;22(21):11697. 10.3390/ijms222111697. 10.3390/ijms222111697 PMC8584273 34769126

[62] Rocco AB, Levalley JC, Eldridge JA, Marsh SA, Rodgers BD. A novel protocol for assessing exercise performance and dystropathophysiology in the *mdx* mouse. Muscle Nerve. 2014;50(4):541–8. 10.1002/mus.24184. 24449511 10.1002/mus.24184

[63] Schindelin J, Arganda-Carreras I, Frise E, Kaynig V, Longair M, Pietzsch T, Preibisch S, Rueden C, Saalfeld S, Schmid B, Tinevez J-Y, White DJ, Hartenstein V, Eliceiri K, Tomancak P, Cardona A. Fiji: an open-source platform for biological-image analysis. Nat Methods. 2012;9(7):676–82. 10.1038/nmeth.2019. 22743772 10.1038/nmeth.2019 PMC3855844

[64] Sheehan DW, Birnkrant DJ, Benditt JO, Eagle M, Finder JD, Kissel J, Kravitz RM, Sawnani H, Shell R, Sussman MD, Wolfe LF. Respiratory Management of the Patient With Duchenne Muscular Dystrophy. Pediatrics. 2018;142(Suppl 2):S62–71. 10.1542/peds.2018-0333H. 30275250 10.1542/peds.2018-0333H

[65] Shih JA, Folch A, Wong BL. Duchenne Muscular Dystrophy: the Heart of the Matter. Curr Heart Fail Rep. 2020;17(3):57–66. 10.1007/s11897-020-00456-0. 32270339 10.1007/s11897-020-00456-0

[66] Sheremeta CL, Yarlagadda S, Smythe ML, Noakes PG. Prostaglandins in the Inflamed Central Nervous System: Potential Therapeutic Targets. Curr Drug Targets. 2024;25(13):885–908. 10.2174/0113894501323980240815113851. 39177131 10.2174/0113894501323980240815113851 PMC11774313

[67] Srivastava M, Baig MS. NOS1 mediates AP1 nuclear translocation and inflammatory response. Biomed Pharmacother. 2018;102:839–47. 10.1016/j.biopha.2018.03.069. 29605772 10.1016/j.biopha.2018.03.069

[68] Sun C, Shen L, Zhang Z, Xie X. Therapeutic Strategies for Duchenne Muscular Dystrophy: An Update. Genes (Basel). 2020;11(8). 10.3390/genes11080837 10.3390/genes11080837 PMC7463903 32717791

[69] Takeshita E, Komaki H, Shimizu-Motohashi Y, Ishiyama A, Sasaki M, Takeda S. A phase I study of TAS-205 in patients with Duchenne muscular dystrophy. Ann Clin Transl Neurol. 2018;5(11):1338–49. 10.1002/acn3.651. 30480028 10.1002/acn3.651 PMC6243382

[70] Tanaka K, Aritake K, Tayama M, Sasaki E, Utsugi T, Sasaoka T, Urade Y. G.P.88: Novel inhibitor of hematopoietic prostaglandin D synthase improves the muscle disorder in an experimental model of Duchenne muscular dystrophy. Neuromuscular Disord. 2014;24(9):821. 10.1016/j.nmd.2014.06.102

[71] Theret M, Saclier M, Messina G, Rossi FMV. Macrophages in Skeletal Muscle Dystrophies. An Entangled Partner J Neuromuscul Dis. 2022;9(1):1–23. 10.3233/JND-210737. 34542080 10.3233/JND-210737 PMC8842758

[72] Tolosa L, Donato MT, Gómez-Lechón MJ. General Cytotoxicity Assessment by Means of the MTT Assay. In M. Vinken & V. Rogiers (Eds.), *Protocols in In Vitro Hepatocyte Research* (pp. 333–348). Springer New York. 2015. 10.1007/978-1-4939-2074-7_26 10.1007/978-1-4939-2074-7_26 26272156

[73] Treat-NMD. Treat-NMD SOP's. 2024.Retrieved 15/07/2024 from https://www.treat-nmd.org/resources-and-support/sop-library/mdx-mouse-dmd/

[74] Tripodi L, Villa C, Molinaro D, Torrente Y, Farini A. The Immune System in Duchenne Muscular Dystrophy Pathogenesis. Biomedicines. 2021;9(10). 10.3390/biomedicines9101447 10.3390/biomedicines9101447 PMC8533196 34680564

[75] Tulangekar A, Sztal TE. Inflammation in Duchenne Muscular Dystrophy-Exploring the Role of Neutrophils in Muscle Damage and Regeneration. Biomedicines. 2021;9(10). 10.3390/biomedicines9101366 10.3390/biomedicines9101366 PMC8533596 34680483

[76] van den Engel-Hoek L, Erasmus CE, Hendriks JC, Geurts AC, Klein WM, Pillen S, Sie LT, de Swart BJ, de Groot IJ. Oral muscles are progressively affected in Duchenne muscular dystrophy: implications for dysphagia treatment. J Neurol. 2013;260(5):1295–303. 10.1007/s00415-012-6793-y. 23263593 10.1007/s00415-012-6793-y

[77] Villalta SA, Nguyen HX, Deng B, Gotoh T, Tidball JG. Shifts in macrophage phenotypes and macrophage competition for arginine metabolism affect the severity of muscle pathology in muscular dystrophy. Hum Mol Genet. 2009;18(3):482–96. 10.1093/hmg/ddn376. 18996917 10.1093/hmg/ddn376 PMC2638796

[78] Wang X, Zhou L. The Many Roles of Macrophages in Skeletal Muscle Injury and Repair. Front Cell Dev Biol. 2022;10:952249. 10.3389/fcell.2022.952249. 35898401 10.3389/fcell.2022.952249 PMC9309511

[79] Wang X, Zhou L. The multifaceted role of macrophages in homeostatic and injured skeletal muscle. Front Immunol. 2023;14:1274816. 10.3389/fimmu.2023.1274816. 37954602 10.3389/fimmu.2023.1274816 PMC10634307

[80] Wehling M, Spencer MJ, Tidball JG. A nitric oxide synthase transgene ameliorates muscular dystrophy in *mdx* mice. J Cell Biol. 2001;155(1):123–31. 10.1083/jcb.200105110. 11581289 10.1083/jcb.200105110 PMC2150800

[81] Wynn TA, Vannella KM. Macrophages in Tissue Repair, Regeneration, and Fibrosis. Immunity. 2016;44(3), 450–462. 10.1016/j.immuni.2016.02.015 10.1016/j.immuni.2016.02.015 PMC4794754 26982353

[82] Yamanouchi K, Tanaka Y, Ikeda M, Kato S, Okino R, Nishi H, Hakuno F, Takahashi S-I, Chambers J, Matsuwaki T, Uchida K. Macroglossia and less advanced dystrophic change in the tongue muscle of the Duchenne muscular dystrophy rat. Skeletal Muscle. 2022;12(1):24. 10.1186/s13395-022-00307-7. 36258243 10.1186/s13395-022-00307-7 PMC9580129

[83] Yao S, Chen Z, Yu Y, Zhang N, Jiang H, Zhang G, Zhang Z, Zhang B. (2021). Current Pharmacological Strategies for Duchenne Muscular Dystrophy [Review]. Front Cell Dev Biol. 2021;9. 10.3389/fcell.2021.689533 10.3389/fcell.2021.689533 PMC8417245 34490244

[84] Yarlagadda S, Kulis C, Noakes PG, Smythe ML. Hematopoietic Prostaglandin D Synthase Inhibitor PK007 Decreases Muscle Necrosis in DMD *mdx* Model Mice. Life (Basel). 2021;11(9):994. 10.3390/life11090994. 34575143 10.3390/life11090994 PMC8469723

[85] Yasir M, Goyal A, Sonthalia S. Corticosteroid Adverse Effects. In StatPearls. 2024. https://www.ncbi.nlm.nih.gov/pubmed/30285357 30285357

[86] Zhang T, Kong X. Recent advances of glucocorticoids in the treatment of Duchenne muscular dystrophy (Review). Exp Ther Med. 2021;21(5):447. 10.3892/etm.2021.9875. 33777191 10.3892/etm.2021.9875 PMC7967797

