# Supplementary material for: Pharmacology and macrophage modulation of HPGDS inhibitor PK007 demonstrate reduced disease severity in DMD-affected muscles of the mdx mouse model
Source: Skelet Muscle. 2025 Apr 24;15:11. doi: 10.1186/s13395-025-00379-1 (PMC12020277; doi:10.1186/s13395-025-00379-1)
Supplement: Supplementary file 1 — Supplementary Material 1. [file 13395_2025_379_MOESM1_ESM.docx]

**Supplementary Data**


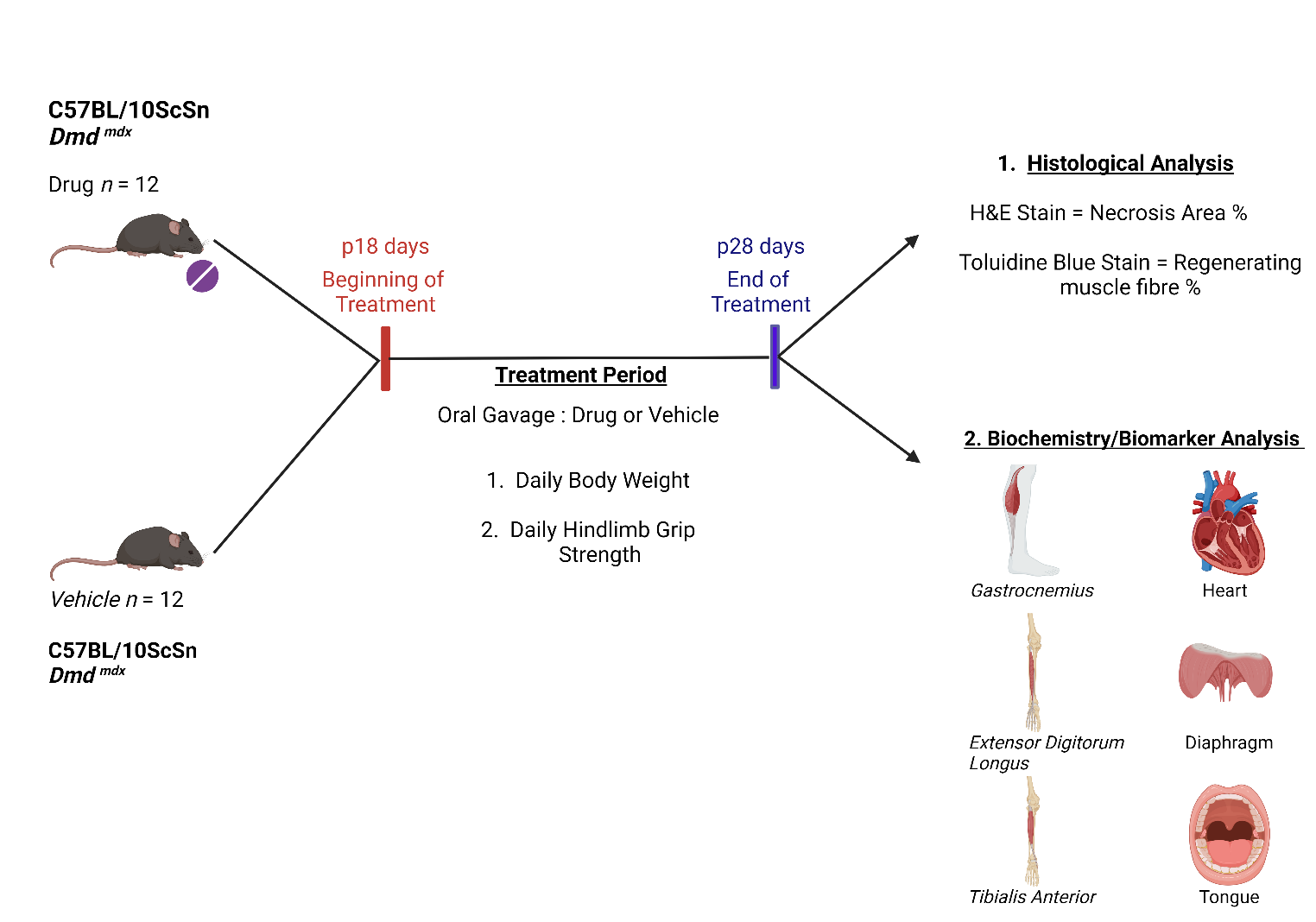


**Supplementary Figure 1: Graphical representation of the study plan assessing PK007 against vehicle-treated acute-stage *mdx* mice (p18-p28 days).** PK007 and Vehicle-treated *mdx* mice were treated daily (oral gavage) in the acute phase over a 10-day treatment period. Daily body weight and hindlimb grip strength were assessed for muscle strength. At the end of the treatment period, mice were euthanised via cervical dislocation. The GA, TA, EDL, heart, diaphragm, and tongue muscles were collected for histochemical stains or biochemistry analysis.


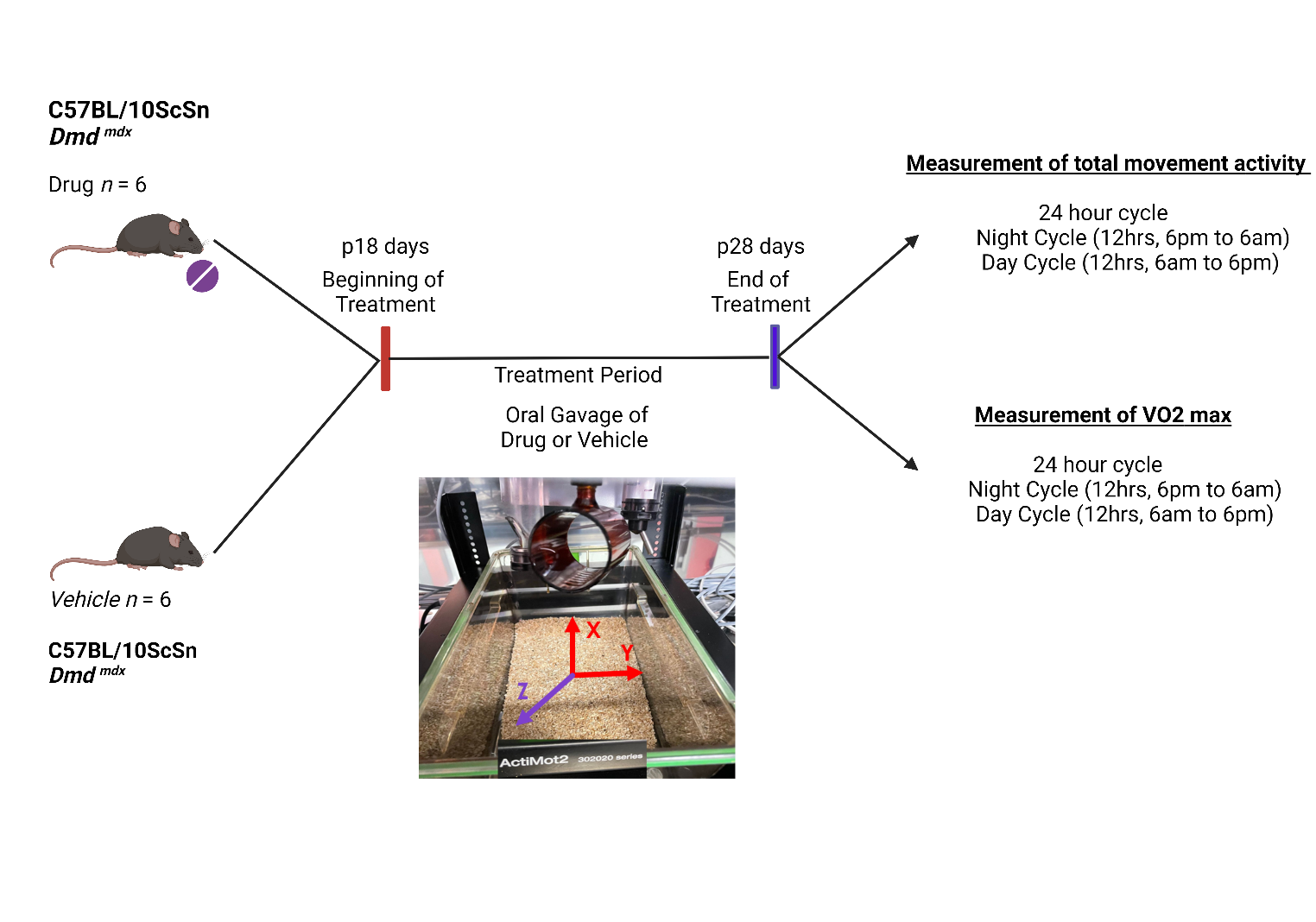


**Supplementary Figure 2: Graphical representation of the study plan assessing PK007 against vehicle-treated acute-stage *mdx* mice (p18-p28 days) in the Phenomaster.** Each mouse was placed in an individual metabolic cage and received daily oral gavage treatment during the acute phase over 10 days. The software measured body weight (when mice rest in the red cylinder), assessed movement through lasers recording movement on the x, y, and z axes hourly for 24 hours over 10 days, and calculated the mice's VO_2_ levels based on their breathing rate within the metabolic cage.


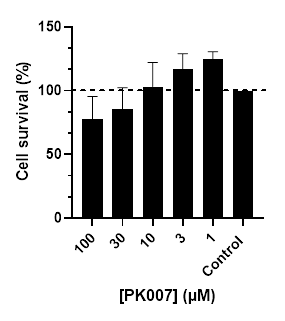


**Supplementary Figure 3: PK007 treatment correlates to high cell survivability.** RAW 264.7 cells were treated with varying concentrations (100 – 1 µM) of HPGDS inhibitor, PK007, and assessed for cell survivability (%). Cell survivability was quantified through an MTT assay; data was collected in triplicates across 4 assays (*n*=4). Data presented as mean±SEM.

**Supplementary Table 1: Pharmacokinetic parameters of PK007**. PK007 was dosed in C57BL/6 mice (*n*=3 for each treatment) orally (PO, 10 mg/kg) and intravenously (IV, 2mg/kg). The sampling time was over 24 hours (hrs), and plasma concentration was measured. Pharmacokinetic evaluations were conducted by WuXi AppTec (Shanghai, China). Data shown includes half-life (T_1/2_), area under the curve (AUC), concentration (C_0_ and C_max_), volume of distribution (steady state, VD_ss_), clearance (Cl), and bioavailability—data displayed as mean ± SEM.

|  | **IV (2 mg/kg)** | | | | | **PO (10 mg/kg)** | | | |
| --- | --- | --- | --- | --- | --- | --- | --- | --- | --- |
| **Name** | **T_1/2_ (hr)** | **AUC_0-last_(h.nM)** | **C_0_ (nM)** | **VD_ss_ (L/kg)** | **Cl (mL/min/kg)** | **T_1/2_ (hr)** | **AUC_0-last_(h.nM)** | **C_max_ (nM)** | **Bioavailability (%)** |
|  | 1.9±0.3 | 276±19.4 | 259±10.1 | 7.30±4.6 | 50.4±4.8 | 3.0±0.3 | 1118±45.0 | 360±16.4 | 81 |

**
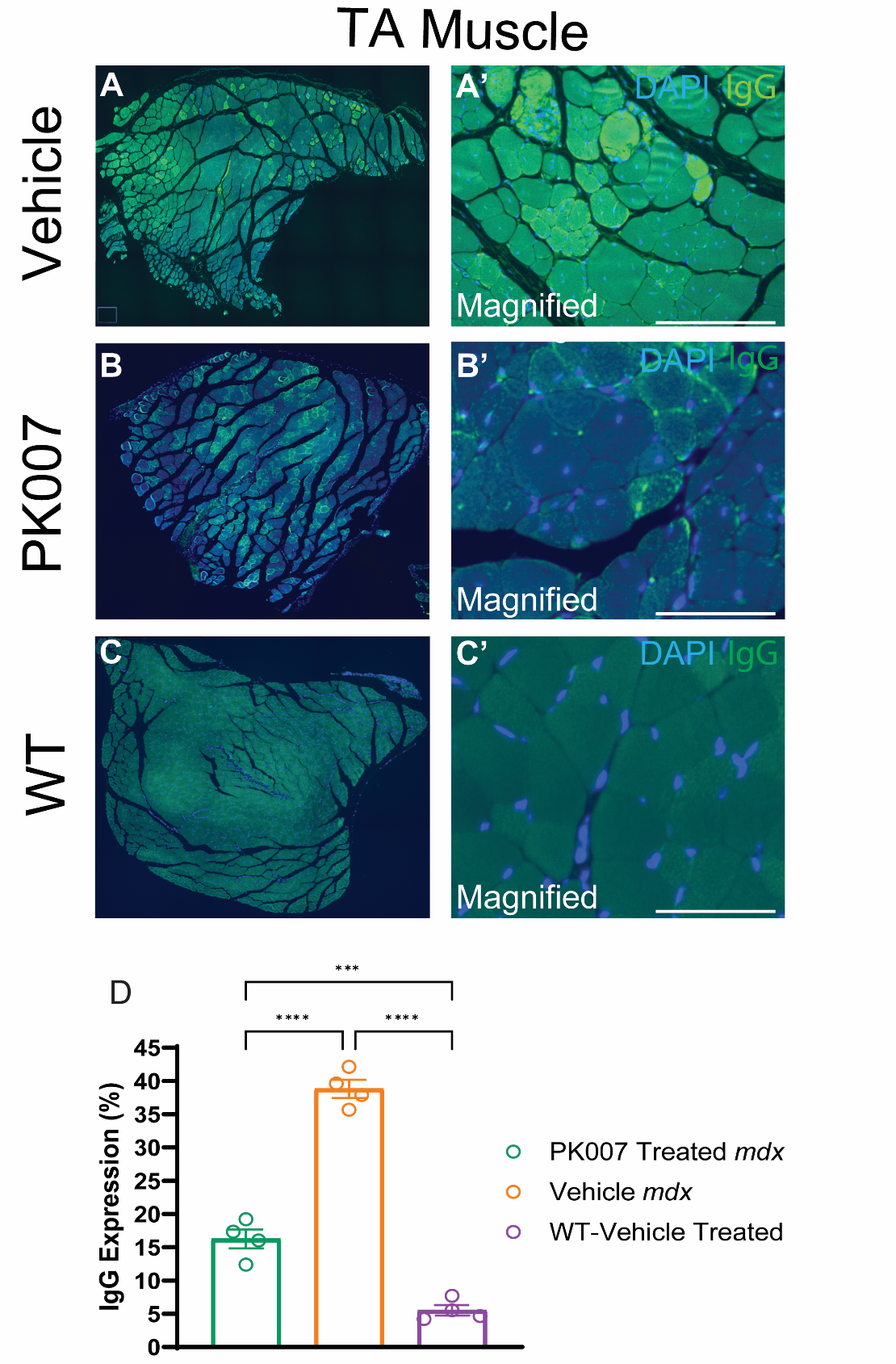
Supplementary Figure 4: PK007 reduces IgG infiltration in tibialis anterior (*mdx*) muscles compared to vehicle treatment.** Transverse sections of TA muscles from vehicle-treated *mdx* (**A, A’**), PK007-treated *mdx* (**B, B’**), and WT-vehicle-treated mice (**C, C’**), stained for IgG infiltration (green) with DAPI-labelled nuclei (blue). Magnified regions (**A’-C’**) highlight areas of IgG infiltration indicative of muscle fibre damage and permeability. PK007-treated *mdx* muscles show reduced IgG-positive regions compared to vehicle-treated *mdx* muscles, with WT muscles as the baseline for minimal IgG infiltration. Scale bars = 10 µm. Quantification of IgG-positive area as a percentage of total muscle area is shown in **panel D**. PK007 treatment significantly reduced IgG expression in *mdx* muscles compared to vehicle-treated controls (p < 0.0001), while IgG infiltration in WT-vehicle-treated muscles was significantly lower than in both vehicle-treated *mdx* muscles (p < 0.0001) and PK007-treated *mdx* muscles (p = 0.0004). Data are presented as mean ± SEM, with individual points representing biological replicates. Statistical significance was determined using one-way ANOVA followed by Tukey's post hoc test.


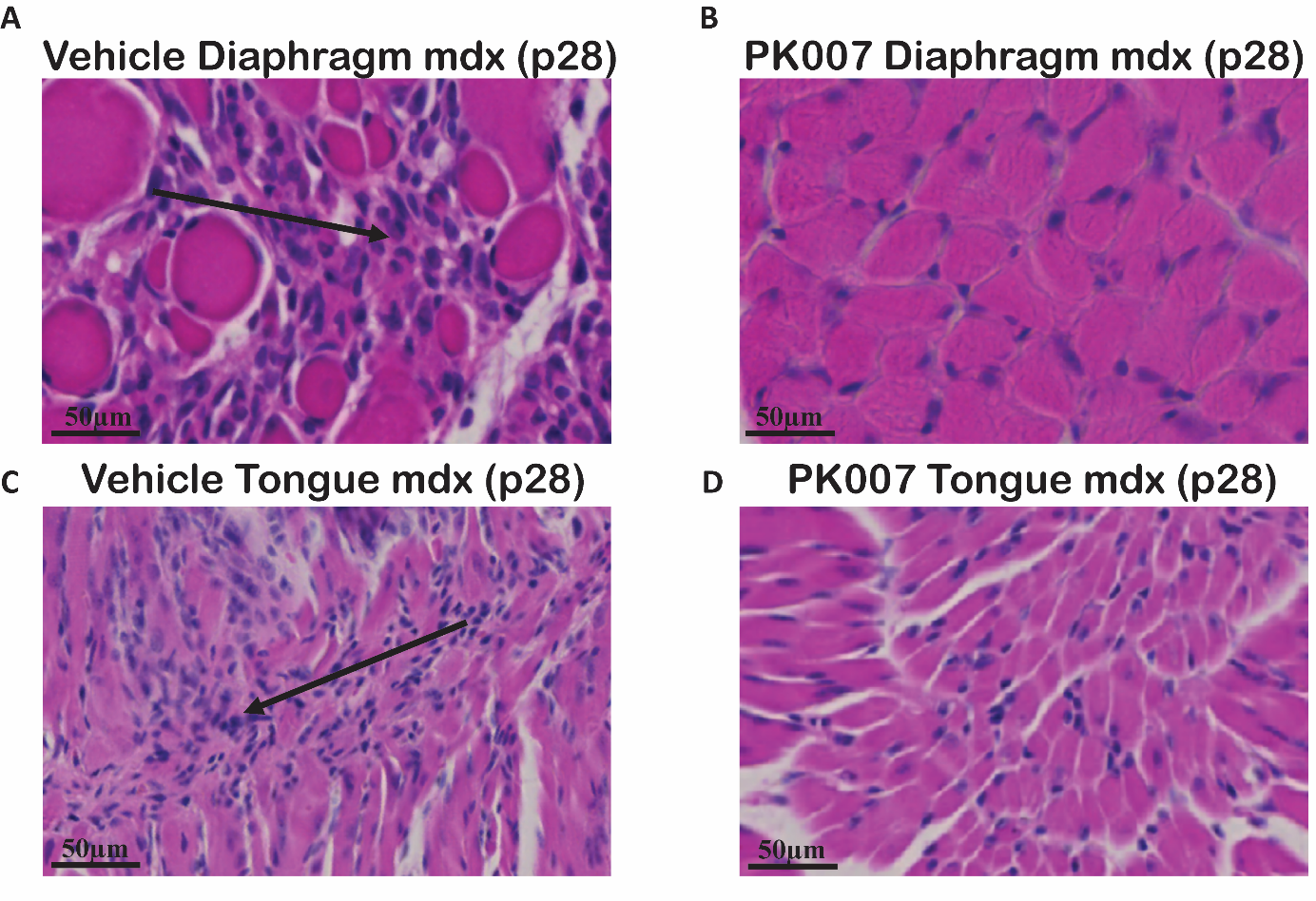


**Supplementary Figure 5**: Myonecrosis is reduced in PK007-treated mdx diaphragm and tongue compared to vehicle-treated mdx mice after 10 days of treatment (postnatal day 28 [p28]). **A** to **D** show representative cross-sectional views of H&E stained diaphragm (**A** & **B**) and tongue (**C** & **D**). A and C are vehicle-treated diaphragm and tongue, respectively, while B and D are PK007-treated diaphragm and tongue. Black arrows depict examples of a myonecrotic area, defined by pale cytoplasm, infiltrating immune cells and lack of muscle fibre structure.
